# Banana Peel Modified with Deep Eutectic Solvents for Pharmaceutical Water Remediation

**DOI:** 10.1021/acsomega.6c01163

**Published:** 2026-06-03

**Authors:** Gabriela Chamorro Gil, João Antonio Tavares Barboza, Sérgio Scherrer Thomasi, Guilherme Max Dias Ferreira, Andrés Felipe Chamorro

**Affiliations:** † Research Group of Electrochemistry and Environment (GIEMA), Faculty of Basic Sciences, 28027 Universidad Santiago de Cali, Cali 760035, Colombia; ‡ Group of Materials, Interface, and Solutions (MatIS), Department of Chemistry, Federal University of Lavras, Campus Universitário, Lavras, MG 37200-900, Brazil; § Research Group in Environmental Chemistry and Clean Technologies (QUATELI), Department of Chemistry, Faculty of Basic and Applied Sciences, 28019 Universidad Militar Nueva Granada, Cajicá 250247, Colombia

## Abstract

Banana peel (BP) has gained attention as a sustainable, low-cost biosorbent for removing pharmaceuticals from aquatic environments. In this study, BP was modified using a choline chloride/methanesulfonic acid (ChCl/MSA) deep eutectic solvent to produce a chemically and structurally enhanced material (BP-ChCl/MSA) for pharmaceutical adsorption from water. The modified biosorbent was comprehensively characterized by FTIR, TGA, and SEM analyses, and its performance was systematically compared with BP treated solely with methanesulfonic acid (BP–MSA). MSA induced holocellulose hydrolysis and ChCl/MSA increased the lignin content in the biosorbent. Propranolol (PRO), metformin, and tinidazole were used as model drugs. BP-ChCl/MSA showed selective adsorption for PRO, with almost 90% of removal efficiency achieved from an initial concentration of 20.0 mg L^–1^, without requiring pH adjustment at 25 °C. The PRO adsorption kinetics for both BP-MSA and BP-ChCl/MSA were best described by the pseudo-second-order (PSO) model, with adsorption occurring in less than 500 min. Isotherms were well described by the Sips model, indicating adsorption on a heterogeneous surface, with BP-ChCl/MSA exhibiting higher maximum adsorption capacity value (284.6 mg g^–1^) than BP-MSA (238.1 mg g^–1^). Thermodynamic analysis using the partition model provided more consistent results, with Δ_ads_ *G* ^◦^ ranging from −24.1 to −21.9 kJ mol^–1^ as the temperature increased from 25 to 40 °C, showing an endothermic process (Δ_ads_H^◦^ = 23.5 kJ mol^–1^) that is entropy-driven (Δ_ads_S^◦^ around 0.15 kJ mol^–1^ K^–1^). Overall, ChCl/MSA treatment enhanced the interactions between the remaining lignin functional groups and PRO, mainly through electrostatic interactions and hydrogen bonding. Moreover, the material showed good reusability, maintaining more than 75% of removal efficiency after 3 adsorption cycles. These results demonstrate that treatment with the ChCl/MSA DES significantly improved the adsorption capacity of BP through an efficient and eco-friendly approach, yielding a sustainable and promising material for drug adsorption in aqueous media.

## Graphical abstract

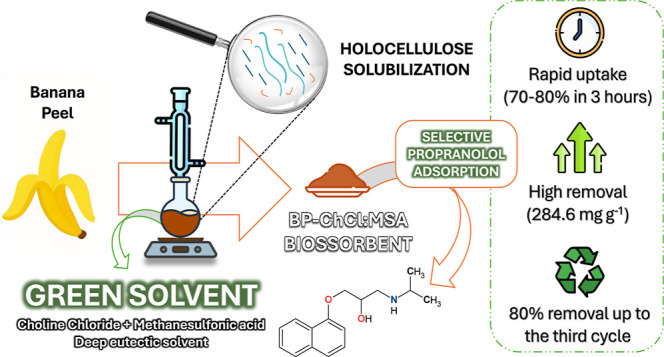

## 1 Introduction

The contamination of aquatic environments by emergent contaminants (EC), particularly those originating from anthropogenic activities, has attracted increasing attention. Among these EC, pharmaceuticals and personal care products have become a major subject of study in recent years due to their negative environmental impacts.^1,2^ In the pharmaceutical industry, these compounds are defined as pharmacologically active substances capable of resisting degradation, remaining persistent in aqueous environments, and, most importantly, exhibiting potential ecological toxicity by causing adverse effects in aquatic species.^3,4^ Despite being designed to physiologically interact with the human body and be metabolized by enzymes, many active pharmaceutical compounds (PhAC) are excreted unchanged. It is estimated that between 10 and 90% of PhAC are eliminated in their original form through urine or feces, resulting in their continuous discharge into domestic and industrial sewage systems.

In addition to human excretion, their release into the environment also occurs during pharmaceutical manufacturing processes and through the improper disposal of unused or expired medicines, which has led to their detection in wastewater, groundwater, and even drinking water.^5^ Additionally, due to their physicochemical properties and stability, PhAC often lack effective degradation pathways, thereby reducing the efficiency of conventional wastewater treatment plants.^6^ Consequently, the inadvertent release of these compounds into the environment, without control or regulation, has led to the bioaccumulation of biotransformed molecules, which is one of the main challenges associated with pharmaceutical contamination.^5^ This situation raises concerns about potential chronic effects on human health, as the long-term ingestion of these compounds through drinking water may have adverse health impacts.^5,7^

The pharmaceutical industry is among the fastest-growing sectors, driven by the increasing prevalence of chronic diseases, which has led to greater consumption of medicines for both preventive and therapeutic purposes. The most used drug classes include antihypertensives, antidepressants, beta-blockers, nonsteroidal anti-inflammatory drugs, and antidiabetics, along with the recurrent use of antivirals and antimicrobials during the SARS-CoV-2 pandemic.^7^ This trend has intensified the occurrence of PhAC in aquatic systems, exacerbating environmental and public health concerns.

Several remediation approaches have been proposed for the removal of pharmaceutical contaminants from water, such as chemical precipitation, reverse osmosis, membrane filtration, coagulation/flocculation, and ion exchange. However, these methods often have disadvantages, including high energy and reagent consumption and the generation of secondary pollutants requiring further treatment. Therefore, the diversity of PhAC in water calls for new mitigation strategies that are not only effective but also technologically and economically sustainable.^8–10^

Adsorption-based water remediation has been the most widely adopted strategy in recent decades to address water contamination, due to its high efficiency, environmental compatibility, experimental simplicity, and broad applicability.^10^ Traditionally, activated carbon is regarded as the most effective conventional adsorbent for the removal of PhAC, followed by synthetic materials such as polymeric resins, zeolites, and aluminosilicates.^11,12^ Many of these materials are produced through surface functionalization to promote favorable interactions between charged PhAC (either positively or negatively) and the chemical groups on the material surface, which limits their application mainly to ionic PhAC and makes the adsorption of neutral compounds a challenge. Furthermore, despite their high performance, the elevated cost of these materials or unsustainable conditions of production restricts their large-scale application, encouraging the search for unconventional, low-cost adsorbents derived primarily from natural sources, commonly referred to as biosorbents.^10,13^

Biosorbents can be derived from wood biomass, animal residues, and, particularly, agricultural waste.^14,15^ Among agricultural residues, banana peel (BP) stands out due to its availability, low cost, and the abundance of lignocellulosic biomass composed of cellulose, hemicellulose, and lignin.^16^ The surface of these matrices contains organic functional groups, particularly carboxyl and hydroxyl groups, that confer biosorption capacity.^17^ However, studies have demonstrated that untreated BP exhibit low selectivity toward PhAC.^18^ To improve its adsorptive properties, lignocellulosic biomass generally requires pretreatment to disrupt its compact structure and reduce or eliminate recalcitrance, thereby enhancing processability.

Physical or chemical functionalization of the lignocellulosic surface is commonly employed. Traditionally, acidic or alkaline methods are mostly used;^19,20^ however, these approaches are often inefficient and environmentally unsustainable. Consequently, recent research has focused on developing pretreatment methods that promote environmental safety and sustainability. In this context, deep eutectic solvents (DES) have emerged as a promising alternative for biomass modification, exhibiting versatility in applications such as carbohydrate conversion and biopolymer dissolution, owing to their strong hydrogen-bonding capabilities.

DES are green solvents composed of a hydrogen-bond acceptor (HBA) and hydrogen-bond donor (HBD), which exhibit melting points lower than those of their individual components.^18^ From a sustainability perspective, DES are considered eco-friendly and have attracted considerable attention because they are inexpensive, biodegradable, biocompatible, and highly tunable in terms of structural design.^21,22^ DES have been applied to remove lignocellulosic components from various bioresidues. For instance, betaine hydrochloride/lactic acid (LA) has been shown to promote hemicellulose removal from *Eucalyptus* wood.^23^ Similarly, choline chloride (ChCl) combined with lactic acid (LA) has been used for lignin removal from maritime pine sawdust,^24^ and sugar cane bagasse.^25^ In contrast, the DES ChCl/glycerol promotes glucose conversion,^25^ indicating that the specific composition of the DES determines its behavior during biomass pretreatment, depending on the chemical composition of the biomass.^21,22^ Additionally, the modulation in the lignocellulosic fraction of the biomass promoted by DES can change the nature and concentration of functional groups in the material surface, improving adsorption.^26–28^ However, the use of DES for development of biosorbents for water remediation has been underexplored.

In this context, a modification pathway for BP was designed using the ChCl/methanesulfonic acid (MSA) DES, with the objective of developing a novel biosorbent (BP-ChCl/MSA) for the removal of PhAC. Propranolol (PRO), metformin (MET), and tinidazole (TN) (Table 1) were selected as model contaminants due to their distinct physicochemical properties, particularly their Log *P* values, which range from negative (TN) to positive (PRO). To better understand the chemical modifications occurring in BP, an additional treatment was carried out using only MSA. The raw material and the resulting biosorbents were characterized using Fourier-transform infrared spectroscopy with attenuated total reflectance (FTIR–ATR), scanning electron microscopy (SEM), thermogravimetric analysis (TGA), and the determination of pH at the point of zero charge (pH_PZC_). Furthermore, the removal efficiency was evaluated under different pH conditions, followed by kinetic and adsorption modeling to elucidate the adsorption mechanism.

**Table 1:**
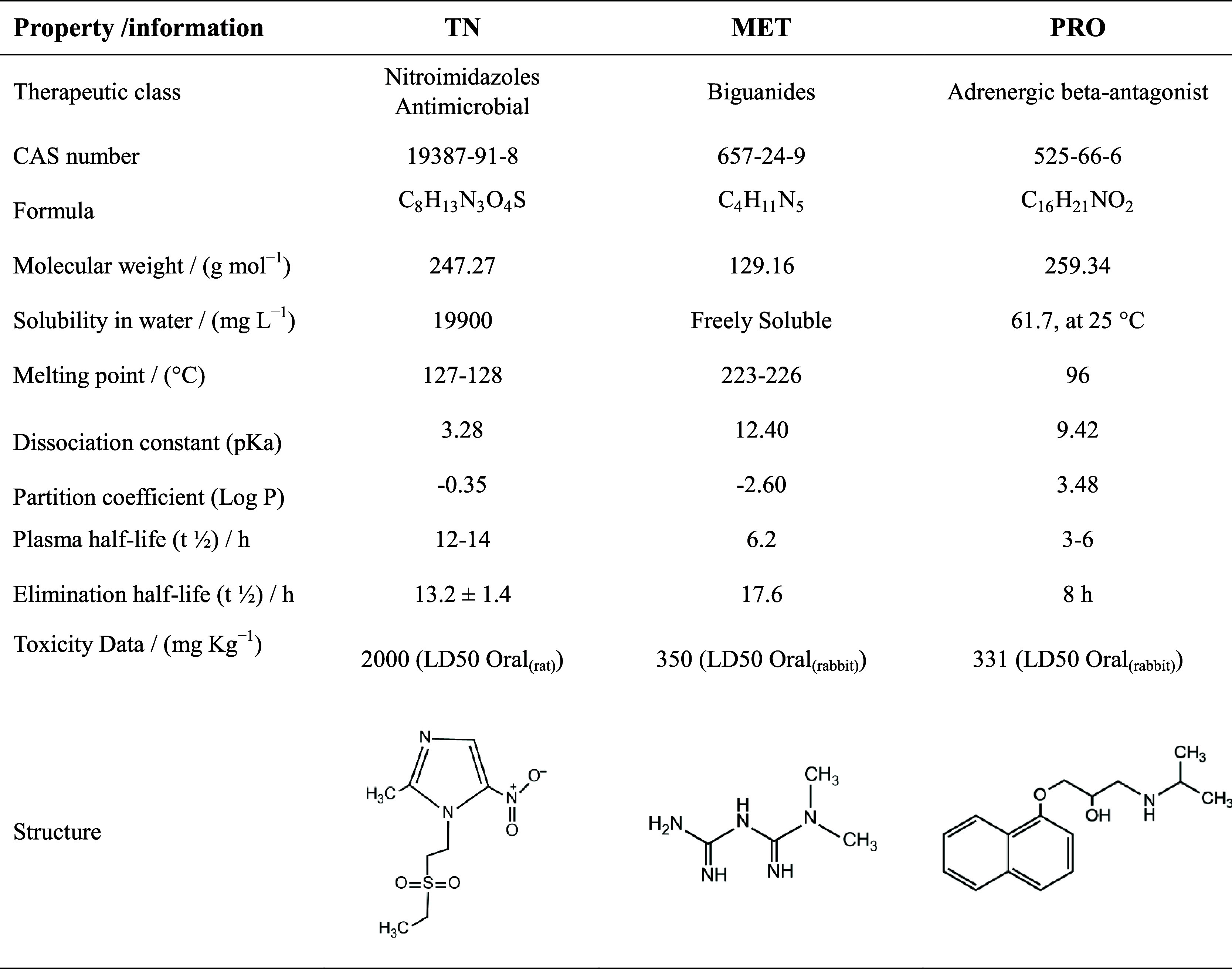
Structures and Properties of TN, MET, and PRO

(Property/information) data obtained from PubChem and DrugBank.^29–32^ .

## 2 Materials and Methods

### 2.1 Materials

Fresh BP were collected from ripe banana (*Musa acuminata*, Cavendish-type) fruits obtained from a local market in Lavras, Brazil. The samples were collected from different batches over several weeks and subsequently homogenized to obtain a representative biosorbent that accounts for the natural variability of the raw material. Methanesulfonic acid (CH_3_SO_3_H, ≥99%) and choline chloride (C_5_H_14_ClNO, ≥98%) were purchased from Sigma-Aldrich. The model drugs propranolol (PRO free base, ≥99%), metformin (MET, ≥98%), and tinidazole (TN, ≥99%) were obtained from Sigma-Aldrich. Deionized water was used throughout all experiments, and all chemicals were employed without further purification.

### 2.2 Preparation of BP Biosorbents

Fresh BP was washed with tap water and deionized water, cut into small pieces, dried in a forced-air oven at 70 °C for approximately 72 h and ground. For BP–MSA preparation, around 15 g of dried BP was treated with a mass ratio of 1.63 g of MSA per gram of dried peel. Specifically, 24.46 g of MSA were dissolved in 200 mL of deionized water, and the resulting solution was transferred to a round-bottom flask containing the preweighed peels. The mixture was heated to approximately 100 °C and maintained under reflux for 24 h. The resulting biosorbent was vacuum-filtered, thoroughly washed with deionized water until the pH of the supernatant approached neutrality, and dried at 70 °C until constant weight. Finally, the material was ground and sieved to a particle size of approximately 0.15 mm. For BP-ChCl/MSA preparation, a DES was synthesized by mixing ChCl (35.53 g) and MSA (24.46 g) at a 1:1 molar ratio in a round-bottom flask. The mixture was stirred on a magnetic plate and heated in a glycerol bath at 50 °C for 2 h, until a clear, viscous liquid was obtained. Subsequently, approximately 15 g of dried BP were added to the DES at a 3:1 DES-to-biomass mass ratio. The mixture was heated to approximately 100 °C and maintained under reflux condensation for 24 h. After treatment, boiling water was added to reduce viscosity, and the material was vacuum-filtered, washed repeatedly with deionized water until neutral pH, and dried at 70 °C to constant weight. The final product was ground and sieved to approximately 0.15 mm particle size.

### 2.3 Physicochemical and Morphological Characterization of Biosorbents

The biosorbents (BP–MSA and BP-ChCl/MSA) were characterized using different techniques. FTIR–ATR was employed to characterize BP, BP-MSA, or BP-ChCl/MSA materials (IR-Affinity^–1^, Shimadzu, Kyoto, Japan). The spectra were collected to evaluate the chemical modification on the biomaterials after reaction, with data acquisition spanning from 4000 to 700 cm^–1^ at 2 cm^–1^ intervals, and 20 scans were conducted per sample. Morphology was evaluated by SEM using a TESCAN CLARA–UHR microscope (Czech Republic). Samples were mounted on aluminum stubs using carbon tape and sputter-coated with carbon prior to analysis. Micrographs were obtained at different magnifications. The TGA was performed on a DTG-60 AH thermogravimetric analyzer (Shimadzu, Japan) using synthetic air at a flow rate of 50 mL·min^–1^. Approximately 10 mg of BP, BP–MSA, or BP-ChCl/MSA were heated from 25 to 900 °C at a heating rate of 10 °C·min^–1^. The pH_PZC_ was determined by mixing approximately 0.025 g of each biosorbent with 25.0 mL of 0.100 mol L^–1^ NaCl aqueous solution. The initial pH of each NaCl aqueous solution was adjusted within the range of 2–12 using a benchtop pH meter (MS TECNOPON mPA210). Dispersions were agitated for 24 h, and the final pH values were recorded. The pH_PZC_ was defined as the point at which the variation of pH (ΔpH) vs initial pH (pH_0_) curve intersected the zero line. This experiment was performed in duplicate.

### 2.4 Biosorption Experiments

#### 2.4.1 Batch Experimental Procedure

The adsorption capacity of BP-MSA and BP-ChCl/MSA was evaluated using PRO, MET, and TN as model drugs. Approximately 10 mg of biosorbent were added to 10.00 mL of a drug solution (20.0 mg L^–1^), and shaken at 100 rpm for 24 h at 25 °C until equilibrium was reached. All PRO solutions were prepared in an ethanol–water mixture (10% v v^–1^ ethanol) to enhance the solubility of the compound. Afterward, the mixtures were centrifuged at 6000 rpm, and an aliquot of the supernatant was collected and analyzed in a UV–visible spectrophotometer (600 Plus, Merck, Darmstadt, Germany). Equilibrium concentrations of PRO, MET, and TN were determined at wavelengths of 289, 234, and 317 nm,^33–35^ respectively. The PRO was selected for next adsorption experiments.

The effect of the initial pH on PRO adsorption was investigated using 20.0 mg L^–1^ solutions of PRO, whose pH values were adjusted to 2, 4, 7, and 10 with HCl (1 mol L^–1^) or NaOH (1 mol L^–1^) solutions. In addition, the effect of the dose of biosorbent was evaluated using 0.5, 1, 1.5, or 2 g L^–1^ of BP-MSA or BP-ChCl/MSA. The removal efficiency (%RE) was calculated according to eq 1

1 $$\%RE=\frac{C_{0}-C_{f}}{C_{f}}\times 100$$

where *C*_0_ and *C*_*f*_ are the initial and final concentrations of the adsorbate (mg L^–1^), respectively.

#### 2.4.2 Kinetic and Equilibrium Studies

Kinetic experiments were performed by mixing approximately 10 mg of BP-MSA or BP-ChCl/MSA with 10.00 mL of a 20.0 mg L^–1^ PRO solution prepared in an ethanol–water mixture (10% v v^–1^ ethanol), and pH = 7. The suspensions were agitated at 100 rpm at 25 ± 2 °C for contact times ranging from 60 to 1440 min. Subsequently, the supernatants were collected, centrifuged, and analyzed using a UV–Visible spectrophotometer, as previously described. The amount of substance adsorbed (*q*_*e*_), in mg g^–1^, were calculated using eq 2

2 $$q_{e}=\frac{C_{0}-C_{f}}{m}V$$

where *C*_0_ and *C*_*f*_ are the initial and final (after a certain time t of contact, which can be or not at the equilibrium condition) concentrations of adsorbate in the supernatant (mg L^–1^), m is the mass of adsorbent (g), and V is the volume (L) of solution added.

The kinetic data were fitted to the nonlinear forms of the pseudo-first-order (PFO),^36^ pseudo-second-order (PSO),^37^ Elovich,^38^ and intraparticle diffusion^39^ models (eqs 3–6)­

3 $$q_{t}=q_{e}\left(1-e^{-k_{1}t}\right)$$

4 $$q_{t}=\frac{q_{e}^{2}k_{2}t}{1+q_{e}k_{2}t}$$

5 $$q_{t}=\frac{1}{\beta }\ln\left(1+\alpha \beta t\right)$$

6 $$q_{t}=k_{dif}t^{1/2}+C$$

where *k*_1_ (min^–1^) and *k*_2_ (g mg^–1^ min^–1^) are the rate constants of the PFO and PSO models, respectively, β (mg g^–1^) and α (mg g^–1^ min^–1^) are the desorption constant and the initial adsorption rate, respectively, of the Elovich model, *k*_dif_ (mg g^–1^ min^–0^·^5^) is the intraparticle diffusion coefficient, and *C* (mg g^–1^) is the constant related to diffusion resistance.

Adsorption isotherms were obtained by mixing approximately 10 mg of BP-MSA or BP-ChCl/MSA with 10.00 mL of PRO solutions (pH = 7) at initial concentrations ranging from 5 to 1500 mg L^–1^. The mixtures were agitated at 100 rpm and maintained at 25 ± 2 °C for 24 h. Subsequently, the supernatants were collected, centrifuged, diluted when necessary, and analyzed by UV–Vis spectrophotometry, and *q*_*e*_ was calculated using eq 2. The equilibrium data were modeled using the nonlinear Freundlich,^40^ Langmuir,^41^ and Sips^42^ isotherms (eqs 7–9)­

7 $$q_{e}=K_{f}C_{e}^{1/n}$$

8 $$q_{e}=\frac{q_{\max}K_{L}C_{e}}{1+K_{L}C_{e}}$$

9 $$q_{e}=\frac{q_{\max}{K_{s}C_{e}}^{n_{s}}}{1+{K_{s}C_{e}}^{n_{s}}}$$

where *K*_*L*_ (L mg^–1^) is the Langmuir equilibrium constant, 1/*n* and *K*_*f*_ (mg g^–1^) (L mg^–1^)^1/n^ are the Freundlich adsorption capacity constant and the constant related to surface heterogeneity, respectively. $K_{S}\;{\left(L\;mg^{-1}\right)}^{n_{s}}$ is the Sips constant and n_S_ is the parameter to describe the heterogeneity of the surface of the adsorption system. Finally, *q*_max_ (mg g^–1^) is maximum amount of PRO adsorbed.

The performance of each model was evaluated using the adjusted coefficient of determination (*R* ^2^_adj_), the reduced chi-square (χ_red_^2^), and the agreement between the experimental and theoretical *q*_*e*_ values. All experiments were conducted in triplicate, with coefficients of variation below 14.5%.

#### 2.4.3 Biosorption Thermodynamics

Biosorption experiments were carried out at different temperatures (25, 30, 35, and 40 °C) by mixing approximately 10 of BP-MSA or BP-ChCl/MSA with 10.00 mL of a 20.0 mg L^–1^ PRO solution (prepared in an ethanol–water mixture, 10% v v^–1^ ethanol). The suspensions were agitated at 100 rpm for 24 h at the designated temperature. After equilibration, the supernatants were collected, centrifuged, and analyzed using a UV–Vis spectrophotometer. All experiments were performed in triplicate.

### 2.5 Reusability and Selectivity Assays of BP-ChCl/MSA

Four adsorption cycles of PRO on BP-ChCl/MSA were performed. For each cycle, approximately 10 mg of BP-ChCl/MSA was mixed with 10.0 mL of a 20.0 mg L^–1^ PRO solution, prepared in an ethanol–water mixture (10% v v^–1^ ethanol), at pH 7 and stirred at 100 rpm at 25 ± 2 °C for 24 h. Afterward, the sample was treated as described in Section 2.4.1 to calculate the %RE (eq 1). PRO was desorbed using 10 mL of ethanol (96%) and stirred for 24 h. The system was then centrifuged, and the supernatant was removed to reuse the material in cycle 2. This procedure was repeated until four adsorption cycles were completed, and all experiments were performed in triplicate.

Selectivity toward PRO adsorption was evaluated in the presence of common interfering species typically encountered in aqueous environments. Batch experiments were conducted by mixing approximately 10 mg of BP-ChCl/MSA with 10.0 mL of a 20.0 mg L^–1^ PRO solution prepared in salt media at pH 7, using an ethanol–water mixture (10% v v^–1^ ethanol) as the solvent. The suspensions were agitated at 100 rpm and maintained at 25 ± 2 °C for 24 h. The inorganic salts investigated included NaCl, KCl, K_2_HPO_4_, and Na_2_CO_3_, evaluated individually or as a mixed system containing all salts at a concentration of 0.1% (m v^–1^). After equilibration, the residual PRO concentration was determined as described in Section 2.4.1, and the %RE was calculated according to eq 1. The adsorption procedure was further assessed in the presence of 20.0 mg L^–1^ MET and in tap water to better simulate realistic sample matrices. All experiments were performed in triplicate.

## 3 Results and Discussion

### 3.1 Materials Production and Characterization

To understand the chemical modifications of BP by the ChCl/MSA DES, ATR–FTIR spectra of BP, BP-MSA, and BP-ChCl/MSA were recorded (Figure 1).

**Figure 1:**
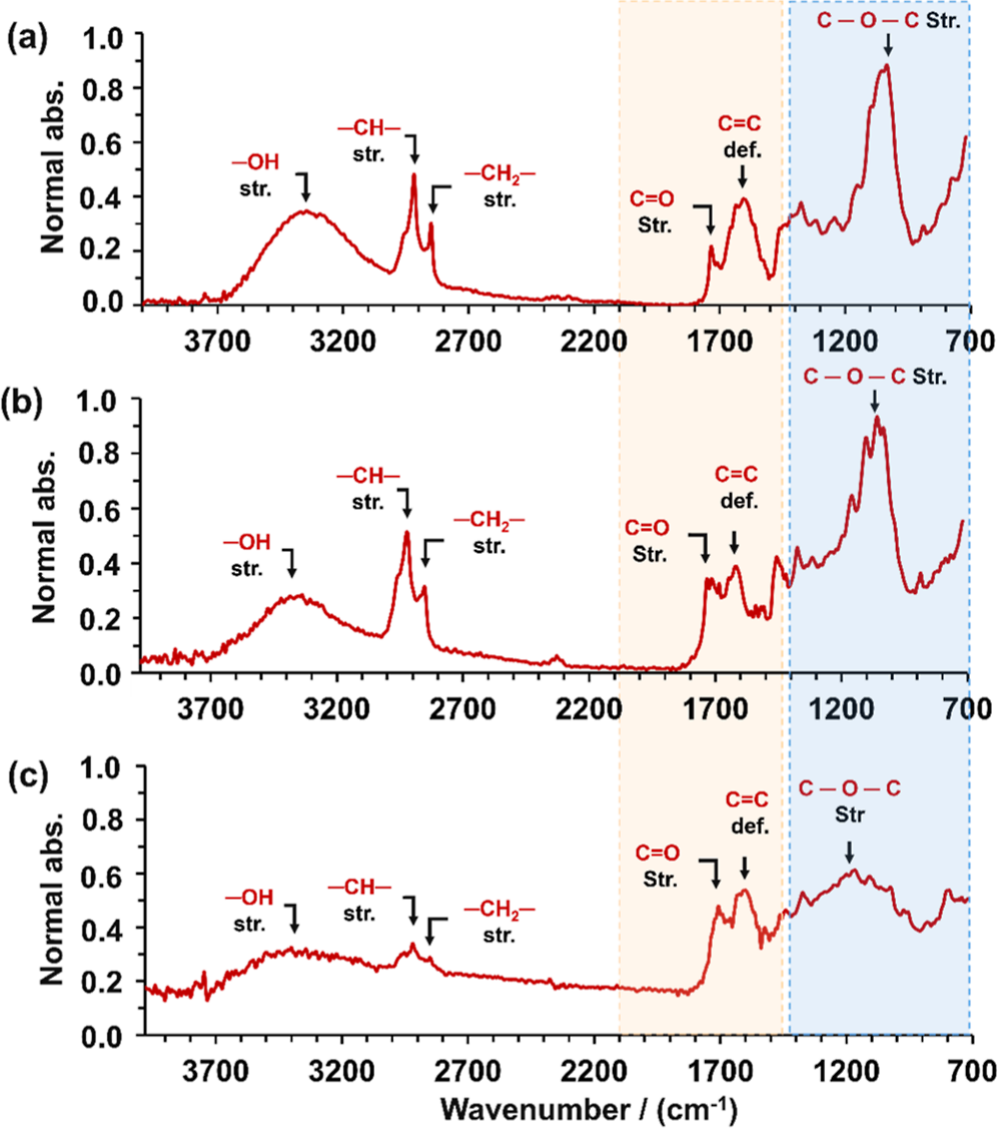
ATR–FTIR spectra of: (a) BP, (b) BP-MSA and (c) BP-ChCl/MSA. Spectra were normalized.

In the spectrum of BP, the characteristic bands of polysaccharide structures such as cellulose and hemicellulose can be observed, with notable bands at 3356 cm^–1^, 2919 cm^–1^, and 2840 cm^–1^, attributed to the stretching vibrations of O–H and to the asymmetric and symmetric stretching vibrations of aliphatic C–H bonds present in the glucose units. Additionally, a characteristic band at 1030 cm^–1^ appears, corresponding to the C–O stretching vibrations and the symmetric stretching of C–O–C bonds within the glucose ring and in the glycosidic linkages in cellulose.^16,43^ A strong band at 1730 cm^–1^ is also observed, which is assigned to the stretching vibrations of the carbonyl-containing groups in hemicellulose, confirming the presence of this polysaccharide in the biomaterial, or partial disruption of lignin-carbohydrate complexes, possible oxidation processes, and/or structural rearrangements in lignin. Finally, the band at 1595 cm^–1^ corresponds to the C=C stretching, arising from the aromatic ring vibrations associated with the guaiacyl (G) and syringyl (S) units of lignin. These bands are consistent with those previously reported for BP samples.^44^

BP-MSA spectrum shows the same characteristic bands as that of BP, with a prominent band at 1030 cm^–1^, indicating that the glucose units were not removed by MSA. However, when comparing the BP and BP-MSA spectra, overlapping of the bands between 1600 cm^–1^ and 1500 cm^–1^ can be observed, along with an increase in the intensity of the band at 1716 cm^–1^, attributed to carbonyl-containing groups. This effect is likely due to chemical interactions between MSA and the biopolymer, which can alter matrix compaction or modify the lignocellulosic fractions. Under the reaction conditions used for BP modification, sulfonation of the polysaccharides present in the matrix did not occur, as strong sulfating agents such as sulfuric acid or sulfamic acid are required to achieve effective sulfonation.^44,45^ Nevertheless, it is hypothesized that partial hydrolysis and depolymerization of cellulose and hemicellulose in BP occurred, leading to the exposure of carboxylic and carbonyl groups in the matrix (see TGA analysis below).

The spectral profile of BP-ChCl/MSA changes markedly, particularly with the alteration of the bands corresponding to the symmetric stretching of C–O–C bonds, which lose resolution. The precision in identifying the band maxima was affected by the high noise-to-signal ratio. In addition, the bands attributed to the carbonyl-containing groups of hemicellulose and the C=C of lignin, at 1701 cm^–1^ and 1611 cm^–1^, respectively, exhibit increased resolution and higher intensity compared with that in BP-MSA spectrum. Conversely, the intensity of the band corresponding to the C–H bonds of the glucose units at 2913 cm^–1^ decreases significantly. All these spectral modifications indicate that treatment with ChCl/MSA alters the polysaccharide structure and composition of the biosorbent. The increase in the carbonyl band suggests the formation and/or enrichment of oxygen-containing functional groups, while the decrease in the C–H band is consistent with a more effective removal of hemicellulose from BP. Because MSA forms hydrogen bonds with ChCl to generate the DES and no water was added to the system, hydrolysis of the biomass likely occurs to a lesser extent in BP-ChCl/MSA. In this scenario, the DES appears to act primarily as an efficient solvent for hemicellulose, promoting its partial solubilization. To corroborate and understand better the hydrolysis and solubilization effects, TGA analyses were performed for BP, BP-MSA, and BP-ChCl/MSA (Figure 2).

**Figure 2:**
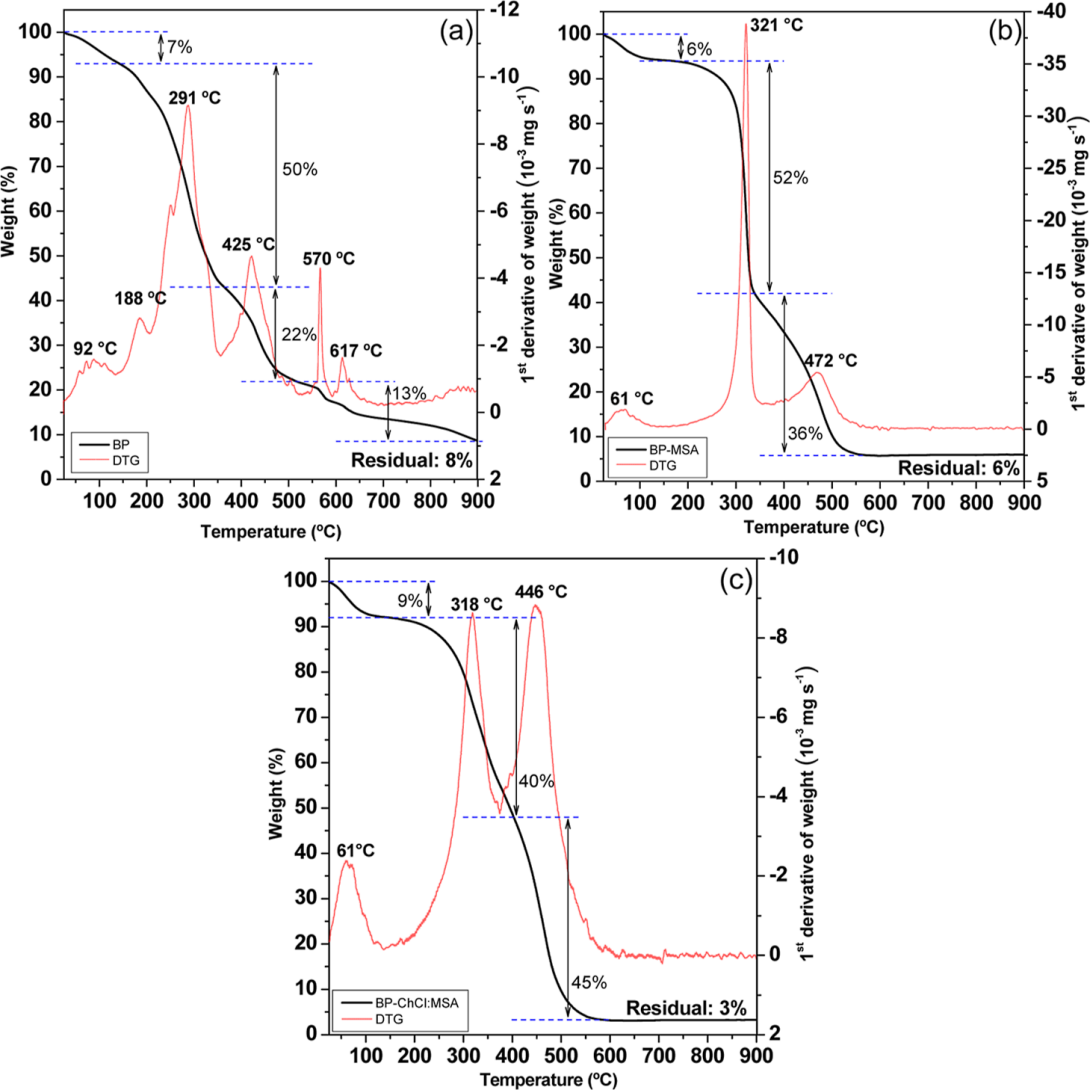
TGA analysis of: (a) BP, (b) BP-MSA, and (c) BP-ChCl/MSA.

For the BP sample, four distinct degradation stages were observed (Figure 2a). The first one occurred from room temperature up to approximately 180 °C and corresponds to the loss of water, pectin, and volatile organic compounds (such as pigments, oils, terpenes, and polyphenols) entrapped in the biopolymeric matrix, accounting for about 7% of the total weight loss.^46,47^ The second degradation stage ranged from approximately 200 to 380 °C, resulting in approximately 50% weight loss, associated with a maximum degradation temperature of 291 °C, and corresponds mainly to the oxidative thermal degradation of hemicellulose and cellulose, and part of lignin, which decomposes slower, over a broader temperature range (200–500 °C).^48^ Similar results were reported for banana residues,^49^ where the decomposition of both hemicellulose and cellulose occurred between approximately 230 to 380 °C. The third degradation stage, occurring between 350 and 480 °C, accounted for a 22% weight loss, with a maximum degradation rate observed at 425 °C. This stage is primarily attributed to the decomposition of lignin.^50^ Finally, a fourth degradation stage occurred above 500 °C, with an additional weight loss of approximately 13%, probably associated with the decomposition of more complex and thermically stable lignin structures that require higher temperatures for degradation.

On the other hand, the BP-MSA and BP-ChCl/MSA samples exhibited three weight-loss stages, demonstrating changes in chemical composition compared with BP. The first stage occurred with a maximum degradation rate of 61 °C for both materials, producing weight losses of 6% and 9%, respectively. These results suggest that all materials retained a hydrophilic character, as they can entrap water, and that the water retention capacity increased when the material was treated with ChCl/MSA. Therefore, these materials are expected to exhibit favorable interactions with pollutants in hydrophilic media. The second degradation stage, occurring between 250 and 350 °C, corresponded to an approximate 52% weight loss for BP-MSA, similar to that observed for BP, and is mainly associated with the thermal degradation of hemicellulose and cellulose. However, the degradation rate was markedly higher, reaching nearly four times that of the unmodified material. This can be attributed to the introduction of carbonyl functional groups (see increase in the carbonyl band at around 1715 cm^–1^ in the BP-MSA spectra), enhanced acidity, and structural depolymerization induced by MSA treatment, which collectively lower the thermal stability of the holocellulose fraction of the biomass.

Interestingly, BP-ChCl/MSA exhibited a 40% weight loss between 250 and 350 °C, 12% smaller compared with BP-MSA, corroborating the hypothesis that MSA induces the partial hydrolysis of cellulose and hemicellulose present in the biomaterial, while treatment with ChCl/MSA promotes a solvent effect that enhances the solubility of these biopolymers. These results are further supported by the observed increase in lignin content in BP-ChCl/MSA, resulting from the loss of cellulose and hemicellulose, with more stable lignin (degraded after 400 °C) contents increasing from around 30% for BP and BP-MSA to 45% for BP-ChCl/MSA.

Additionally, treatment with MSA and ChCl/MSA produced marked morphological changes on the BP surface (Figure 3). SEM micrographs of the BP show that lignocellulosic fibers display a highly rough texture, with fibers adhered to each other due to lignin and other organic linkages, forming a fibrous network with pores and channels. In contrast, the BP-MSA and BP-ChCl/MSA samples show notable surface changes, exhibiting denser and more compact morphologies with fewer like-craters pores and less surface texture than the untreated BP, indicating that the lignocellulosic fibers were disrupted, forming a more homogeneous structure. BP-ChCl/MS appears disintegrated because of the ChCl/MSA treatment, which caused the breakdown of the matrix into fine particles.

**Figure 3:**
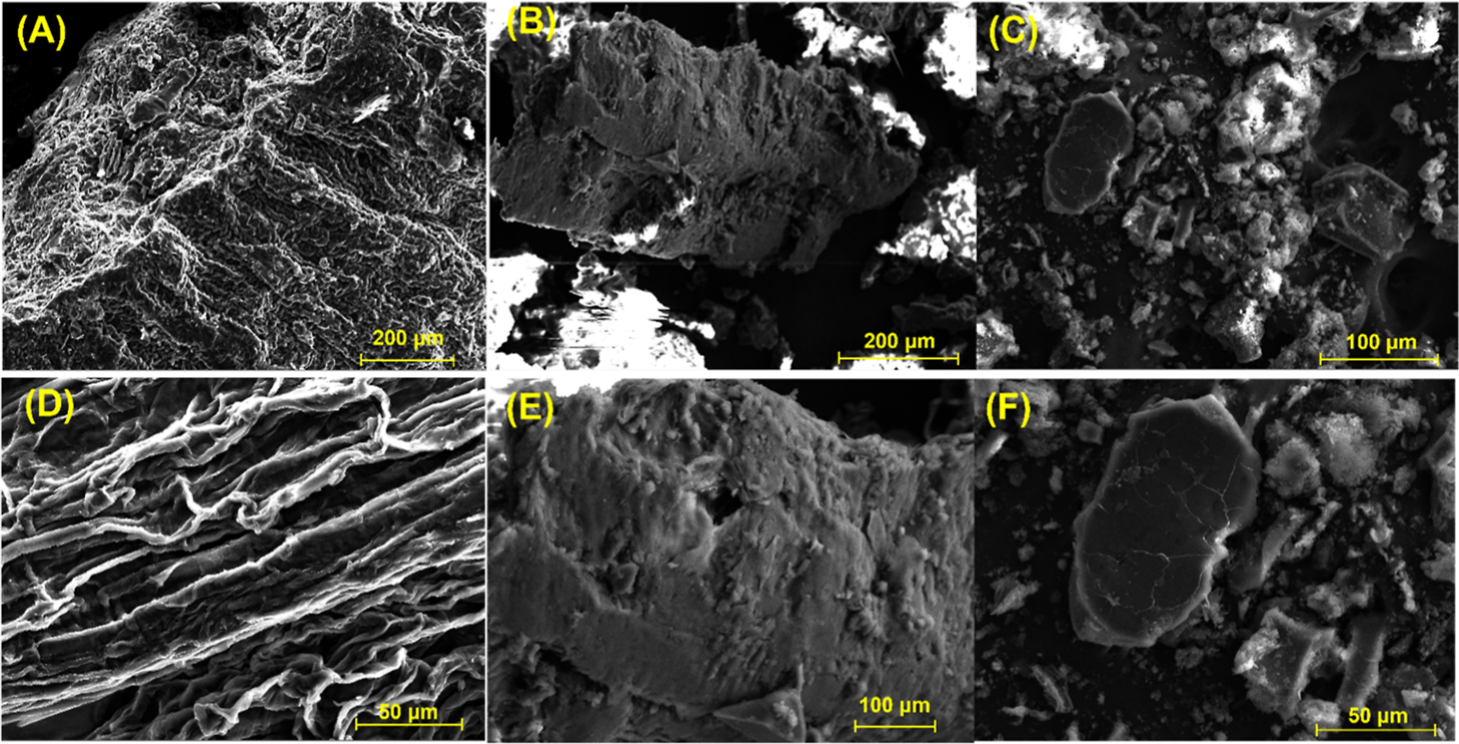
SEM micrographs of: (A, D) BP, (B, E) BP-MSA, and (C, F) BP-ChCl/MSA.

Considering the physicochemical properties observed, plausible mechanistic hypotheses can be proposed to elucidate the factors governing the properties of each material (Figure S1—Supporting Information). According with Mishra et al.,^51^ the Molecular Electrostatic Potential (MEP) of MSA shows the highest negative charge density around the oxygen atoms of the sulfonic acid group. Through hydrogen-bond formation, the acidic proton is transferred from the sulfonic acid group to water, promoting ionization in water and releasing proton and methanesulfonate ions. The oxygen atoms of the β-1,4 linkages in cellulose and hemicellulose can then attack the hydronium ion, inducing the hydrolysis of these polysaccharides. This observation may explain the increase in the degradation rate observed between 250 and 350 °C for BP-MSA in the TGA results.

On the other hand, when BP was treated with ChCl/MSA, hydrolysis reactions were partially hindered due to the low amount of water in the system and the strong interaction between ChCl and MSA in the formation of the DES, in which the chloride anion acts as a HBA. Then, the ChCl/MSA solvent can promote the separation of lignocellulosic components (Figure S2—Supporting Information) due to two main factors: (i) MSA, acting as a HBD, forms hydrogen bonds with hydrophilic groups in cellulose, disrupting its crystalline zones and consequently increasing cellulose dissolution; and (ii) ChCl, acting as a HBA, interacts with electron-deficient groups in the hydroxyl moieties of lignin and hemicellulose, promoting the breakdown of hydrogen bonds and facilitating the dissolution of these biopolymers^52^ (See Figure S3 – Supporting Information). Since the TGA results showed that BP-ChCl/MSA had a higher lignin content (∼48%), it can be concluded that ChCl/MSA promotes greater dissolution of hemicellulose than of lignin.

Overall, the use of MSA or ChCl/MSA DES can modulate the structural properties of BP, enabling the design of biosorbents with tailored functionalities. Importantly, because these treatments act on common structural components of lignocellulosic biomass (cellulose, hemicellulose, and lignin), the observed modifications are expected to occur regardless of the specific variety or source of BP. This suggests that the proposed approach can be broadly applied to different banana residues, supporting the general applicability of DES-based strategies for the development of efficient biosorbents.

### 3.2 Adsorption Assays

Considering the molecular polarity and the predominant functional groups of TN, MET, and PRO, distinct adsorption behaviors can be expected when interacting with biomass-based materials (Table 1). The high polarity and hydrogen-bonding capacity of MET favor interactions with oxygenated and acidic surface sites, whereas the intermediate polarity and amphiphilic nature of PRO enable hydrophobic and π–π interactions with aromatic domains of lignin. In contrast, the presence of nitro and sulfonyl groups in TN confers both polar and electron-withdrawing character, promoting dipole–dipole and electrostatic interactions with surface functionalities. In this context, these compounds can serve as model contaminants to evaluate how the modifications applied to BP enhance its adsorption performance.

Figure 4 illustrates the removal performance of BP, B-MSA, and B-ChCl/MSA materials toward the contaminants MET, TN, and PRO. These contaminants exhibit distinct molecular structures, bearing specific organic functional groups, tunable charges, and varying hydrophobicities, which enable probing the removal potential of the modified materials. While TN exhibited %RE below 5%, MET removal reached values above 15%, with only minor variations among the evaluated materials. In contrast, PRO showed removal efficiencies exceeding 50%, with BP-ChCl/MSA achieving nearly 90%, indicating a remarkable selectivity toward this compound.

**Figure 4:**
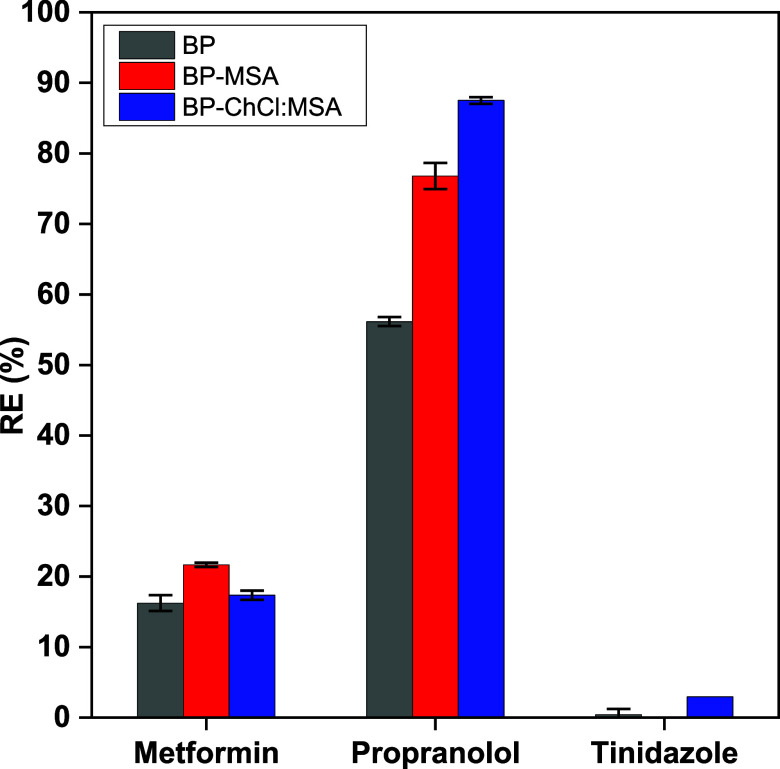
%RE of MET, TN, and PRO using BP, BP–MSA, and BP–ChCl/MSA. Adsorption conditions: 10.0 mg of material +10.00 mL of drug solution (20.0 mg L^–1^), stirred at 100 rpm for 24 h at 25 °C, no adjustment of initial pH.

DES have demonstrated significant potential for the fractionation of lignocellulosic biomass. During this pretreatment, partial dissolution of hemicellulose and/or lignin may occur, which can reduce particle size and potentially increase the accessible surface area of the biomass.^53^ Based on this premise, variations in adsorption performance after DES pretreatment could be attributed, at least in part, to changes in the surface area of the biosorbent.

Although the surface area and pore size distribution were not determined in this study, the potential contribution of surface area changes can be indirectly evaluated. While DES treatment may induce morphological changes such as fragmentation and increased surface irregularity, the SEM images do not reveal the development of well-defined porous structures that would justify a significant increase in surface area. In fact, the treated materials exhibit smoother and more compact surface features, suggesting structural rearrangement rather than pore generation. These observations indicate that DES treatment leads to structural disruption without clear evidence of enhanced porosity.

This interpretation is consistent with the adsorption results shown in Figure 4. Notably, the impact of the DES treatment was not uniform across the evaluated contaminants. A marked improvement in %RE (∼40%) was observed only for PRO relative to BP, whereas MET and TN showed negligible changes in %RE after treatment, with only slight increases in adsorption capacity. Such selective behavior suggests that the improvement in adsorption cannot be attributed to a general increase in surface area or pore development following DES treatment, as these effects would be expected to similarly influence all contaminants. Instead, the absence of a consistent trend indicates that adsorption enhancement is likely influenced by specific interactions between the adsorbates and the surface functional groups introduced or exposed during DES treatment. However, in the absence of quantitative textural characterization (e.g., BET/BJH analysis), the contribution of surface area changes cannot be completely ruled out, and this interpretation should be considered as indirect evidence.

In this context, the enhanced adsorption of PRO may be associated with the presence of less hydrophilic and more organophilic sites on the modified biosorbent surface, favoring stronger hydrophobic and π–π interactions between the aromatic moieties of the molecule and the carbonaceous matrix.

Considering these results and to verify our hypothesis, the adsorption of PRO was further investigated using BP, BP-MSA, and BP-ChCl/MSA. This analysis aimed to provide insights into the specific role of the DES, beyond that of MSA alone, in promoting the formation of favorable adsorption sites for contaminant removal.

#### 3.2.1 pH Effect on PRO Removal

Figure 5a shows the effect of initial pH on the %RE of PRO using different biosorbents (BP, BP-MSA, and BP-ChCl/MSA). The %RE was strongly pH-dependent, with minimal adsorption observed at pH 2 (<10%) and a pronounced increase at higher pH values. For BP, the highest removal was observed at pH 4, followed by a slight decrease at higher pH. In contrast, BP-MSA and BP-ChCl/MSA exhibited maximum removal at pH 7, with both materials showing comparable performance under these conditions. At pH 4, BP-ChCl/MSA showed superior removal performance.

**Figure 5:**
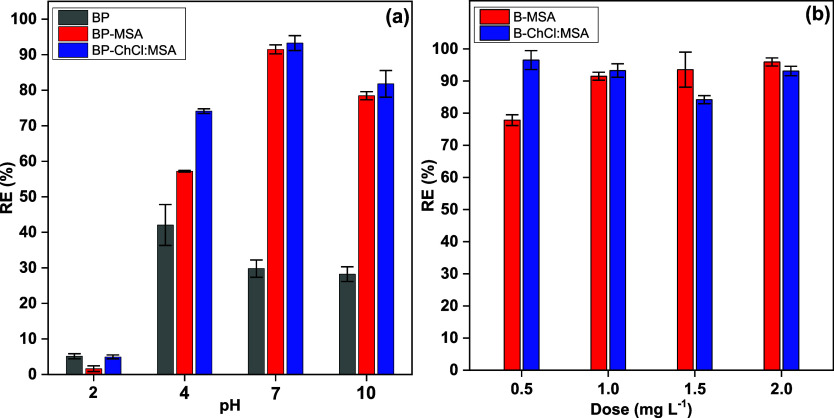
Effect of pH (a) and adsorbent dose (b) on the %RE of PRO. Adsorption conditions for Figure 5a: 10.0 mg of material +10.00 mL of PRO solution (20.0 mg·L^–1^), stirred at 100 rpm for 24 h at 25 °C. For Figure 5b, pH 7 was used to the PRO solution.

PRO is a weak base (pK_a_ ≈ 9.5),^54^ existing predominantly in its cationic form at pH values below approximately 8.5 and gradually shifting toward its neutral form as the pH increases above this. For BP-MSA and BP-ChCl/MSA, the pH_PZC_ are both around 4.5 (Figure S4, Supporting Information), while it is around 7 for BP.^55^ When the solution pH is below the pH_PZC_, the adsorbent surface is predominantly positively charged, whereas at pH values above the pH_PZC_, a net negative surface charge prevails. Accordingly, at pH 2, the low %RE can be attributed to the electrostatic repulsion between the positively charged surface and the protonated drug species, in addition to competition from the high proton concentration for the available adsorption sites.

The pronounced increase in %RE at pH 4 relative to pH 2, observed across all biosorbents, can be reasonably attributed to a reduction in positive surface charge, which decreases electrostatic repulsion and facilitates adsorption. At neutral pH, stronger interactions are expected, primarily due to electrostatic attraction between oppositely charged functional groups on the biosorbents and the protonated PRO. Notably, B-MSA and B-ChCl/MSA maintain %RE values at pH 7 higher than those at pH 4, whereas BP exhibits a marked decline under neutral conditions. This divergence highlights the interplay among multiple dominant removal mechanisms: electrostatic attraction, which is favored within the pH range between the pH of the biosorbent and the pK_a_ of PRO due to the presence of oppositely charged species; hydrogen bonding, which becomes more significant at lower pH values, where protonated functional groups on the adsorbent act as hydrogen bond donors to the electron-rich oxygen sites of PRO; and hydrophobic interactions, which are promoted at pH values near the pH_PZC_, where the surface exhibits reduced net charge.

For BP, the decrease in %RE at neutral pH is likely associated with its higher holocellulose content. The lower density of ionizable oxygen-containing functional groups on BP reduces the surface charge density, thereby weakening electrostatic interactions relative to the modified materials. In addition, the increase in pH may diminish both hydrogen bonding capacity and hydrophobic interactions, contributing to a decline in adsorption efficiency. In contrast, for BP-MSA and BP-ChCl/MSA, strong electrostatic interactions can be established at pH 7, supporting higher removal efficiencies.

These findings suggest that biosorbent composition plays an important role in modulating adsorption mechanisms across different pH conditions. The relative contributions of electrostatic, hydrogen bonding, and hydrophobic interactions are strongly governed by the lignin-to-holocellulose ratio, which ultimately dictates contaminant removal performance. Finally, the decrease in adsorption observed at pH 10 is likely associated with a weakening of electrostatic attraction due to the progressive deprotonation of PRO, further emphasizing the importance of speciation effects (see Section 3.2.6 for additional mechanistic insights).

The observed behavior highlights the critical influence of pH on adsorption performance. Overall, these results demonstrate that surface-functionalized biosorbents exhibit enhanced efficiency under near-neutral pH conditions, particularly BP-ChCl/MSA, confirming their suitability for PRO removal from aqueous media. Importantly, effective performance at neutral pH also reduces operational costs by eliminating the need for pH adjustment. Therefore, subsequent experiments were conducted at pH 7 using only BP-MSA and BP-ChCl/MSA.

#### 3.2.2 Dose Effect on PRO Removal

Figure 5b shows the influence of adsorbent dose on the %RE of PRO for the biosorbents developed in this study. BP-ChCl/MSA achieved consistently high %RE values across the entire dose range, reaching 97% ± 3% removal even at the lowest tested dose (0.5 g L^–1^), highlighting its markedly superior adsorption performance. This behavior indicates that, for BP-ChCl/MSA, the density of accessible adsorption sites was not a limiting factor within the evaluated dose interval. Instead, the high removal at low dose suggests strong and specific interactions between PRO and the active sites, corroborated by the thermodynamic analysis (see Section 3.2.5). From a practical standpoint, this efficiency at low sorbent loadings is particularly advantageous for environmental remediation, as it minimizes material consumption and operational costs without compromising removal performance.

In contrast, B-MSA exhibited 78% ± 1% removal at 0.5 g L^–1^, with %RE increasing steadily as the dose increased, surpassing 95% at 2.0 g L^–1^. This trend reflects the lower molecular affinity of PRO for the polymeric biosorbent under the studied pH conditions. The observed enhancement in removal with increasing dose is therefore attributable mainly to the greater overall surface area and the higher number of available adsorption sites provided by higher sorbent loadings,^44^ rather than to intrinsically strong interactions at the sorbent–contaminant interface.

In summary, the data supports that the engineered biosorbents offer outstanding PRO removal under varying pH and dose conditions, ensuring reliable performance across a spectrum of operational scenarios. These findings underscore the versatility and practical applicability of both materials for water treatment in PRO removal.

#### 3.2.3 Kinetic Studies

Figure 6 depicts the adsorption kinetics of PRO onto BP-MSA and BP-ChCl/MSA. Both materials demonstrate rapid uptake, reaching approximately 70%–80% of the equilibrium adsorption capacity within the first 3 h. This kinetic profile suggests two distinct adsorption regimes: an initial fast phase dominated by the availability of abundant surface sites and strong adsorbate–adsorbent interactions, followed by a slower approach to equilibrium governed by a reduction in available active sites and a gradual diffusion of PRO molecules into the internal matrix of the biosorbent.

**Figure 6:**
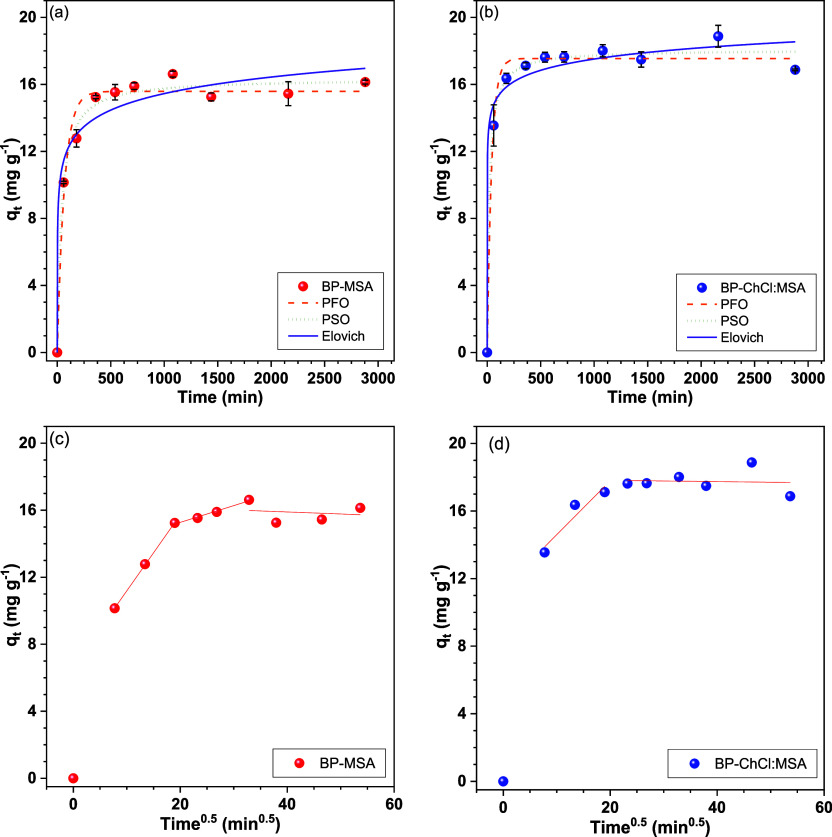
PRO adsorption kinetics and kinetic models evaluated for (a) BP-MSA and (b) BP-ChCl/MSA. (c,d) Intraparticle diffusion model for each adsorbent. Adsorption conditions: 10.0 mg of material +10.00 mL of PRO solution (20.0 mg·L^–1^) at pH 7, stirred at 100 rpm for 24 h at 25 °C.

The kinetic parameters derived from the PFO, PSO, and Elovich models (Table 2) indicate that both biosorbents exhibit adsorption behavior more consistent with the PSO model, as evidenced by higher coefficients of determination (*R* ^2^_adj_ ≥ 0.9875) and lower χ_red_^2^ values (≤0.308 mg^2^ g^–2^). BP-ChCl/MSA exhibited a slightly higher adsorption capacity (18.1 mg g^–1^) and rate constant (*k*_2_ = 2.82 × 10^–3^ g mg^–1^ min^–1^) compared with BP-MSA, confirming that the DES treatment improved surface reactivity and adsorption kinetics. In summary, both BP-MSA and BP-ChCl/MSA offer efficient PRO uptake, with BP-ChCl/MSA exhibiting superior kinetics. These results demonstrate the practical feasibility of using chemically modified banana pseudostem derivatives as biosorbents for rapid and effective PRO removal from aqueous matrices.

**Table 2:** Fitting Parameters of Kinetic Models Describing the Adsorption Behavior of PRO Onto BP-MSA and BP-ChCl/MSA Biosorbents

parameters	biosorbent	
	BP-MSA	BP-ChCl/MSA
***q***_***t***_ (exp)/(mg g^–1^)	16.1	18.9
	pseudo-first order	
***q***_***t***_ (theo)/(mg g^–1^)	15.6	17.5
*k*_1_/min^–1^	1.50 × 10^–2^	2.40 × 10^–2^
*R* ^2^_adj_	0.9727	0.9857
**χ**_**red**_^2^ */*(mg^2^ g^–2^)	0.696	0.445
	pseudo-second order	
***q***_***t***_ (theo)/(mg g^–1^)	16.4	18.1
*k*_2_/(g mg^–1^ min^–1^)	1.61 × 10^–3^	2.82 × 10^–3^
*R* ^2^_adj_	0.9875	0.9907
**χ**_**red**_^2^ */*(mg^2^ g^–2^)	0.308	0.290
	Elovich	
**α**/(mg g^–1^ min^–1^)	73.9	6.61 × 10^4^
**β**/(g mg^–1^)	0.703	1.03
*R* ^2^_adj_	0.9581	0.9746
**χ**_**red**_^2^ */*(mg^2^ g^–2^)	1.07	0.791
	intraparticle diffusion	
*K*_*d*_ */* (mg g^–1^ min^–0.5^ *)*	0.454	0.318
*C /* (mg g^–1^)	6.64	11.4

The PFO and Elovich models exhibited inferior fits, along with inconsistencies in the fitted parameters. In particular, the unusually high α value obtained from the Elovich model for BP-ChCl/MSA is likely overestimated and lacks clear physicalmeaning.

The intraparticle diffusion analysis revealed distinct mass-transfer regimes, as evidenced by the multilinear behavior of the *q*_*t*_ versus *t* ^0.5^ plots. This pattern supports a mechanism involving an initial rapid adsorption phase, followed by a slower intraparticle diffusion stage. In both systems, the nonzero intercepts (*C* ≠ 0) indicate that intraparticle diffusion is not the sole rate-controlling step; external film diffusion or surface adsorption also contributes to the overall kinetics. The BP-MSA system exhibited a higher intraparticle diffusion constant (*K*_*d*_ = 0.454 mg g^–1^ min^–0.5^) and a smaller intercept (*C* = 6.64 mg g^–1^), indicating faster pore diffusion and a reduced boundary-layer effect compared to the PRO system on BP-ChCl/MSA. This agrees with the SEM images, where the BP-MSA apparently showed a more porous surface than BP-ChCl/MSA.

#### 3.2.4 Isotherms

Figure 7 shows the equilibrium adsorption capacity as a function of equilibrium concentration. Both materials exhibit a steep, nearly vertical increase in *q*_*e*_ at low *C*_*e*_ values, characteristic of a high-affinity isotherm, indicating strong interactions between the adsorbate and the biosorbent surface. As *C*_*e*_ increases, the adsorption capacity continues to rise without reaching a clear plateau (higher concentration were not evaluated due to the limit of PRO solubility). The overall shape of the isotherms corresponds to favorable adsorption behavior, classified as *L*-type according to Giles’ classification.^56^

**Figure 7:**
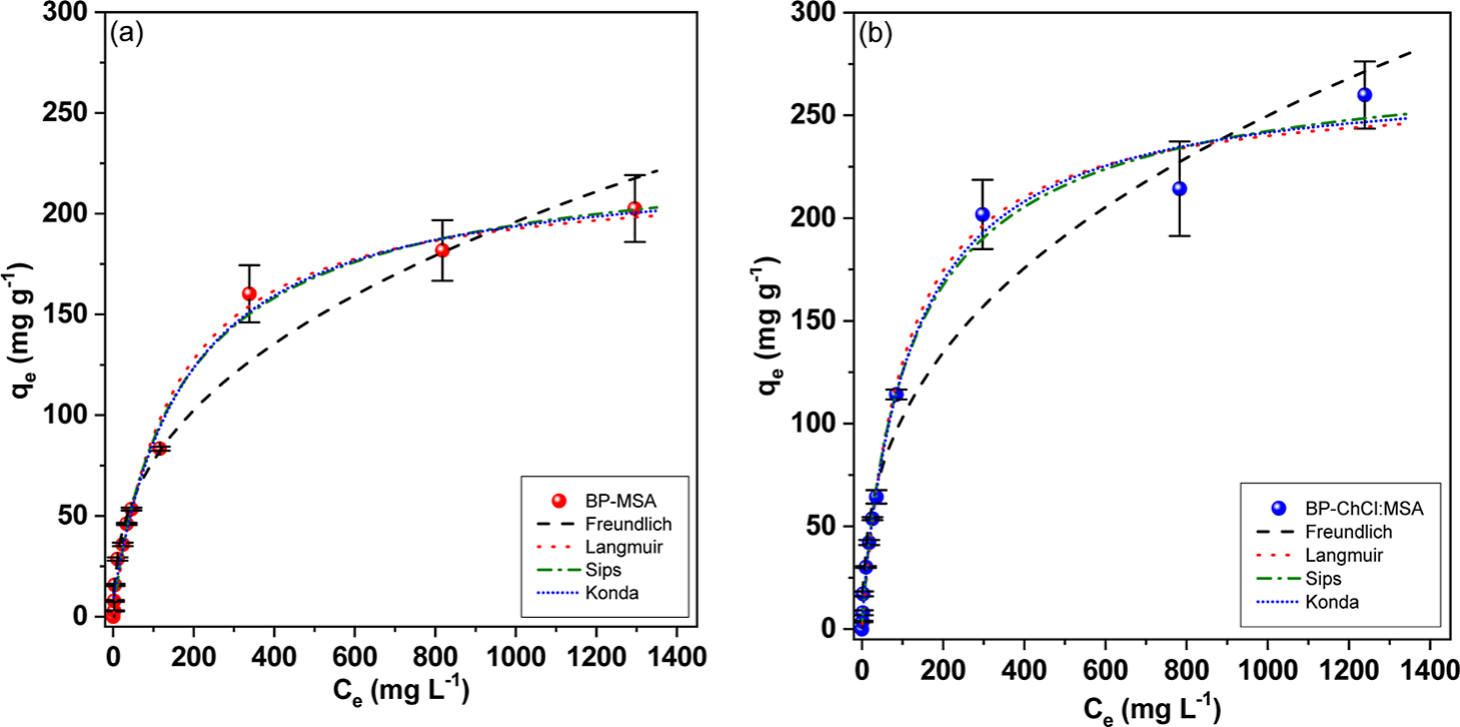
PRO adsorption on (a) BP-MSA and (b) BP-ChCl/MSA. Adsorption conditions: 10.0 mg of material +10.00 mL of PRO solution at pH 7, stirred at 100 rpm for 24 h at 25 °C.

At low *C*_*e*_ (<4 mg L^–1^), both adsorbents display similar *q*_*e*_ values; however, at higher concentrations, BP-ChCl/MSA exhibits a markedly higher adsorption capacity, reaching approximately 260 mg g^–1^ at the highest *C*_*e*_ evaluated. In contrast, BP-MSA shows a lower experimental maximum adsorption capacity around 200 mg g^–1^.

The equilibrium data were fitted to three isotherm models (Freundlich, Langmuir, and Sips), and the corresponding parameters are summarized in Table 3. Among them, the Sips model provided the best fit (*R* ^2^_adj_ > 0.99, χ_red_^2^ ≤ 84.4), indicating a predominantly heterogeneous surface. BP-ChCl/MSA exhibited a higher maximum adsorption capacity (*q*_max_ = 284.6 mg g^–1^, Sips model) compared to the B-MSA (238.1 mg g^–1^), confirming the enhanced adsorption performance resulting from improved surface functionality and interaction energy. The *n*_*s*_ values (>0.88), approaching unity, suggest a low degree of surface heterogeneity, which is further supported by the good fit of the Langmuir model (*R* ^2^_adj_ > 0.9881, **χ**_**red**_^2^ ≤ 88.1). It is noteworthy that the maximum adsorption capacity predicted by the Langmuir model followed the same trend as that observed for the Sips model (*q*_max_ BP-ChCl/MSA > *q*_max_ BP-MSA); however, the adsorption capacity derived from the Sips model was considered more representative of the system due to its superior fit to the experimental data.

**Table 3:** Isotherm Parameters for the Adsorption of PRO Onto BP-MSA and BP-ChCl/MSA Biosorbents Derived from Freundlich, Langmuir, and Sips Models

parameters	biosorbent	
	BP-MSA	BP-ChCl/MSA
*q*_max_ (exp)/(mg g^–1^)	202.5	259.9
	Freundlich	
*K*_*F*_/(mg g^–1^)	12.1	17.5
*n*	2.48	2.60
*R* ^2^_adj_	0.9672	0.9583
**χ**_**red**_^2^ */* (mg^2^ g^–2^)	174	350
	Langmuir	
*q*_max_/(mg g^–1^)	220.4	264.9
*K*_*L*_/(L mg^–1^)	6.89 × 10^–3^	9.62 × 10^–3^
*R* ^2^_adj_	0.9881	0.9895
**χ**_**red**_^2^ */* (mg^2^ g^–2^)	62.8	88.1
	Sips	
*q*_max_/(mg g^–1^)	238.1	284.6
*K*_*s*_/(L mg^–1^)^***n***^_***s***_	1.01 × 10^–2^	1.48 × 10^–2^
*n*_*s*_	0.8811	0.8622
*R* ^2^_adj_	0.9912	0.9900
**χ**_**red**_^2^/(mg^2^g^–2^)	46.5	84.4

Table 4 shows the *q*_*e*_ values of various materials reported in the literature and compares them with those obtained in this research for PRO removal from water. BP-MSA and BP-ChCl/MSA exhibited higher *q*_*e*_ values compared with several materials such as kiwi peels, multiwalled carbon nanotubes, and even activated biochar, which is typically characterized by its high porosity. Only the alginate-derived biomass from brown seaweed and the magnetic cellulose-chitosan nanocomposite showed higher *q*_*e*_ values than BP-ChCl/MSA. However, alginate extraction requires several steps, including treatment with toxic solvents such as formaldehyde.^54^ On the other hand, cellulose-chitosan nanocomposites represent an excellent strategy to increase the surface area of the adsorbent; however, large-scale production of nanomaterials may limit their practical application. In contrast, BP-ChCl/MSA was obtained from bioresidues through a single reaction, in which the DES solvent can be easily recovered, reducing production costs and making these materials a biodegradable and biocompatible alternative for removing drugs such as PRO from aqueous systems.

**Table 4:** Adsorption Capacity of PRO on BP-MSA and BP-ChCl/MSA Compared with Various Adsorbents

sorbent/method	***q***_***e***_ ^a^ (mg g^–1^)	model	temperature (°C)	pH	ref.
BP-MSA	238.1	Sips	25	7.0	present study
BP-ChCl/MSA	284.6	Sips	25	7.0	present study
kiwi peels	2.0	Langmuir	25	5.5	^57^
magnetic graphene oxide and magnetite nanocomposite	16.82	Sips	25	6.0	^58^
multiwalled carbon nanotubes modified functionalized with Fe nanoparticles	78.3^b^	Langmuir	25	6.0	^59^
activated carbon of stone of *Bactris guineensis*	121.4	Langmuir	25	8.0	^60^
activated biochar of fruit peels of *Bactris guineensis*	161.3	Liu	45	8.0	^61^
magnetic cellulose-chitosan nanocomposite	313	Langmuir	-	7.0	^62^
residual biomass from alginate extraction from brow seaweed (*Sargassum filipendula*)	503.1^a^	Langmuir	25	8.5	^63^

a *q*_*e*_ values estimated from model fitting.

b Values converted from mmol g^–1^ to mg g^–1^.

#### 3.2.5 Thermodynamic Analysis

The effect of temperature on PRO adsorption onto BP-ChCl/MSA is shown in Figure 8. As the temperature increased from 25 to 40 °C, the equilibrium adsorption capacity (*q*_*e*_) increased across nearly the entire concentration range studied. This trend indicates that the adsorption process is endothermic.

**Figure 8:**
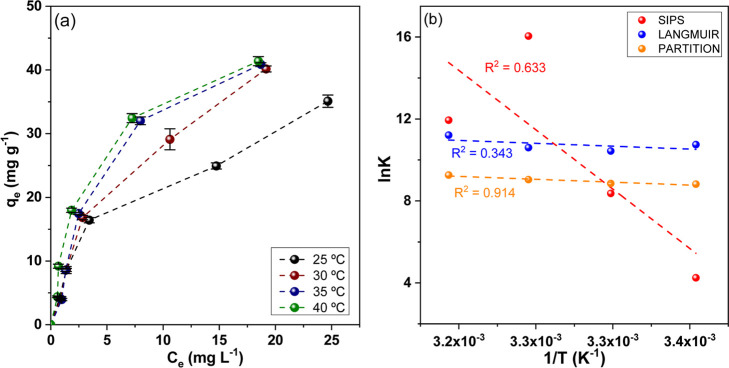
(a) PRO adsorption on BP-ChCl/MSA at different temperatures; (b) Van’t Hoff plot obtained from different models. Adsorption conditions: 10.0 mg of biosorbent +10.00 mL of PRO solution at pH 7, stirred at 100 rpm for 24 h.

The determination of the standard equilibrium constant (*K*_ads_^θ^) for the adsorption process, which is used to determine thermodynamic parameters of adsorption, has been recently discussed in literature, and many inconsistencies have been identified,^64^ especially regarding its dimensionless. Chen et al.^65^ has discussed reasonable calculation of this parameter from adsorption equilibrium constant that has been evaluated here for different isotherm models.

In summary, eq 10 adapted from Liu (2009)^66^ is adopted for the calculation of *K*_ads_^θ^

10 $$K_{ads}^{\theta }=\frac{K_{model}}{\gamma_{e}}\cdot S^{\theta }$$

where γ_e_ is the activity coefficient of the adsorbate at equilibrium, and *K*_model_ is the experimental equilibrium constant, associated with a clear adsorption equation, obtained from an adequate thermodynamic isotherm model, that should be converted to the standard equilibrium constant *K*_ads_^θ^ by evaluating the standard states, summarized in the term *S* ^θ^.

The good agreement of the Sips isotherm with the experimental adsorption data (Figure 7) can justify the use of ${K_{model}=K}_{S}$ to determine *K*_ads_^θ^, in accordance with eq 11

$$K_{ads}^{\theta }=\frac{K_{S}}{\gamma_{e}}{\left(1\;{mol\;L}^{-1}\right)}^{n}$$ 11

where *K*_*S*_, expressed as ${\left({L\;mol}^{-1}\right)}^{n}$ , was obtained from the original *K*_*S*_ in ${\left({L\;mg}^{-1}\right)}^{n}$ (Table 3) by converting the mass unit (mg) in the isotherm data to molar unit (mol). This parameter represents the Sips equilibrium constant associated with the generalized adsorption reaction described in eq 12

12 $$S+nA⇌SA_{n}$$

in which one adsorbate molecule (*A*) interacts with 1/*n* adsorption sites (*S*).^65^ The term ${\left(1\;{mol\;L}^{-1}\right)}^{n}$ is a dimensionless factor that accounts for the standard-state concentration, and *n* describes the degree of surface heterogeneity in the Sips model.

The activity coefficient of PRO can be estimated using the Davies approximation (eq 13),^67^ derived from the extended Debye–Hückel

13 $$\ln\gamma_{e}=-0.5z^{2}\left(\frac{\sqrt{\mu }}{1+\sqrt{\mu }}‐0.3\sqrt{\mu }\right)$$

In this equation, *z* is the charge of the adsorbate species, assumed to be +1 at pH of 7.0, and μ (mol L^–1^) is the ionic strength of the solution, that was obtained considering the concentration of PRO at equilibrium. The Davies equation is considered valid for ionic strengths up to 0.5 mol L^–1^.

The standard Gibbs free energy change for adsorption (Δ_ads_ *G* ^θ^) at temperature T (K) was obtained from eq 14, while enthalpy (Δ_ads_ *H* ^θ^) and entropy (Δ_ads_ *S* ^θ^) adsorption changes were obtained from eqs 15 and 16, respectively

14 $$\Delta_{ads}G^{\theta }=-RTlnK_{ads}^{\theta }$$

15 $$\frac{{d\ln K}_{ads}^{\theta }}{d\left(1/T\right)}=-\frac{\Delta_{ads}H^{\theta }}{R}$$

16 $$\Delta_{ads}G^{\theta }=\Delta_{ads}H^{\theta }-T\Delta_{ads}S^{\theta }$$

in which *R* is the universal constant of the gases.

The thermodynamic parameters obtained for the adsorption process of PRO using the Sips model are summarized in Table 5.

**Table 5:** Thermodynamic Parameters of Adsorption of PRO on BP-ChCl/MSA Obtained from Different Models

model	*K*_ads_^θ^	Δ_ads_ *G* ^θ^	Δ_ads_ *H* ^θ^	Δ_ads_ *S* ^θ^ (kJ·mol^–1^·K^–1^)
		(kJ·mol^–1^)		
Sips *R* ^2^ = 0.633	7.00 × 10^1^	–10.5	482	1.65
	4.30 × 10^3^	–21.1		1.66
	9.30 × 10^6^	–41.1		1.70
	1.53 × 10^5^	–31.1		1.64
Langmuir *R* ^2^ = 0.343	4.66 × 10^4^	–26.6	23.5	0.17
	3.40 × 10^4^	–26.3		0.16
	4.01 × 10^4^	–27.2		0.16
	7.38 × 10^4^	–29.2		0.17
Partitioning model *R* ^2^ = 0.914	6.75 × 10^3^	–21.9	23.5	0.15
	6.97 × 10^3^	–22.3		0.15
	8.43 × 10^3^	–23.2		0.15
	1.05 × 10^4^	–24.1		0.15

The negative values of Δ_ads_ *G* ^θ^ (ranging from −10.5 to −41.1 kJ·mol^–1^) indicates that the adsorption is favored in the equilibrium state under the studied conditions, for each temperature. According to the Sips isotherm model, the adsorption of PRO onto BP-ChCl/MSA is governed by a heterogeneous surface interaction (reaction represented in eq 12).

The heterogeneity factor *n* account for nonuniform energy distributions on the adsorbent surface. Values of *n* <1 indicate surface heterogeneity and the presence of sites with different adsorption energies, while *n* = 1 reduces the model to the homogeneous Langmuir form. Interestingly, *n* >1 values are occasionally reported in literature and are commonly associated with cooperative adsorption phenomena, in which the binding of one molecule enhances the affinity of neighboring sites, or with complex multilayer or aggregation effects at higher adsorbate concentrations.^68,69^ In this study, *n* increased from 0.565 at 25 °C to 1.44 at 35 °C, suggesting that higher temperatures promote a more homogeneous adsorption environment or enhanced interaction between adsorbed molecules. However, when the temperature was further raised to 40 °C, *n* decreased to 1.05, indicating a partial loss of cooperativity and a tendency toward Langmuir-type (monolayer) adsorption behavior.

It is worth noting that when the Sips model was applied to obtain *K*_ads_^θ^, the van’t Hoff plot exhibited poor linearity within the temperature range of 25–45 °C (*R* ^2^ = 0.633). Moreover, its thermodynamic prediction, suggesting a decrease in *K*_ads_^θ^ as the temperature increased from 35 to 40 °C, contradicts the experimental findings, which clearly show an increase in adsorption capacity (Figure 8a) and, consequently, in thermodynamic favorability. This result could indicate that at higher temperatures, the adsorption mechanism may change due to alterations in the surface morphology, pore structure, or functional groups of the adsorbent, consistent with abrupt changes in the *n* parameter. In addition, partial decomposition, desorption of surface-bound species, or rearrangement of active sites could modify the adsorption energetics and disrupt the temperature invariance assumed by the Van’t Hoff equation. This may contribute to the loss of linearity observed in the ln *K* versus 1/*T* plot.

The Δ_ads_ *H* ^θ^ value from van’t Hoff approach remarkably high (482 kJ·mol^–1^) further indicates that the Sips-based thermodynamic estimation does not provide a reliable physical description of the system to provide reasonable thermodynamic interpretation. This high Δ_ads_ *H* ^θ^ means a highly endothermic process, implying that the adsorption would be favored at higher temperatures and could involve significant structural rearrangements or bond formation at the adsorbent surface. The positive Δ_ads_ *S* ^θ^ values (1.64–1.70 kJ·mol^–1^·K^–1^) corroborates the endothermic character, reflecting increased randomness in the system configuration, likely due to the release of solvent molecules and reorganization of surface functional groups.

Considering this, a complementary thermodynamic analysis, incorporating alternative assumptions, can offer valuable insights that purely statistical criteria, used in isotherm analysis, may fail to capture. Indeed, the best-fitting isotherm model does not always yield reliable thermodynamic parameters and may lead to misinterpretation of experimental data. For instance, Jenli et al.^70^ reported that although the Sips equation provided the best statistical fit for the adsorption of cyclohexane carboxylic acid onto a sunflower seed hull cross-linked β-cyclodextrin composite, its thermodynamic consistency was limited. Considering these aspects, two additional adsorption models were evaluated for PRO adsorption: the Langmuir and the partitioning models. The Langmuir model was included due to its good fit for PRO adsorption on BP-ChCl/MSA, while the partitioning model was selected because it provides a simple approach for estimating thermodynamic parameters at low equilibrium concentrations, even when nonlinear isotherms are obtained for adsorption in complex systems.

The thermodynamic analysis based on the Langmuir model assumes the following chemical equation

17 S+A⇌SA

in which each adsorbate molecule interacts with a single adsorption site,^41^ allowing the determination of the thermodynamic constant according to eq 18

18 $$K_{ads}^{\theta }=\frac{K_{L}}{\gamma_{e}}\left(1\;{mol\;L}^{-1}\right)$$

where *K*_*L*_, expressed in L mol^–1^, is the constant obtained from the Langmuir model. The term (1 mol L^–1^) is a dimensionless factor that accounts for the standard-state concentration. Using the constant from eq 18, the thermodynamic parameters were determined according to eqs 14–16 and are summarized in Table 5. As observed, when comparing the linearity of the van’t Hoff plots obtained from the thermodynamic constants of the Langmuir and Sips models, the Sips model exhibited superior linearity.

The partitioning model assumes the process described in eq 19

19 A(aq)⇌A(ads)

which considers the adsorption process as the distribution of adsorbate between solid and aqueous phases, with *K*_*P*_ (partition constant) defined as in eq 20 ^65^

20 $$K_{P}=\frac{q_{e}}{C_{e}}\frac{\left(1\;{mol\;L}^{-1}\right)}{\left(1\;mol\;kg^{-1}\right)}$$

The use of molar units in eq 20 is required to define the equilibrium constant in terms of thermodynamic standard states (1 mol L^–1^ for the aqueous phase and 1 mol kg^–1^ for the adsorbent phase), ensuring that the resulting *K*_P_^°^ is dimensionless and suitable for thermodynamic analysis.

Considering that the adsorption process did not show linear behavior, the partitioning approach can be assumed to be valid only in the limit as *C*_*e*_ → 0, as described in eq 21

$$K_{ads}^{\theta }=\underset{C_{e}\to 0}{\lim}\left[K_{P}\frac{1}{\left(1\;L\;{kg}^{-1}\right)}\right]$$ 21

where the γ_*e*_ was disregarded because the limit consider no solute–solute interaction. The initial slope of the isotherm can be determined from the derivative of an isothermal model as *C*_*e*_ → 0, or alternatively, from other suitable equations that adequately describe the isotherm.^71^ In this work, the experimental isotherms (*q*_*e*_ versus *C*_*e*_, with *q*_*e*_ and *C*_*e*_ in mg g^–1^ and mg L^–1^, respectively) were fitted using exponential eq 22 for temperatures ranging from 25 to 35 °C, and eq 23 for 40 °C

22 $$q_{e}=A_{1}\exp\left(-\frac{C_{e}}{t_{1}}\right)+A_{2}\exp\left(-\frac{C_{e}}{t_{2}}\right)+y_{0}$$

23 $$q_{e}=A_{1}\exp\left(-\frac{C_{e}}{t_{1}}\right)+y_{0}$$

where *A*_1_, *A*_2_, *t*_1_, and *t*_2_ are fitting parameters, and *y*_0_ represents the equilibrium offset. The fitted curves for each model are presented in Figure S5. The *K*_*P*_ values, originally expressed in L g^–1^, were converted to L kg^–1^ prior to their use in the determination of the thermodynamic parameters.

The Van’t Hoff plot obtained from partitioning model (Figure 8b) showed a better linear fit (*R* ^2^ = 0.914) than those derived from the Sips and Langmuir models. This result can be attributed to the *K*_*p*_ value, which reflects the condition of infinite dilution, where the assumption of identical adsorption sites interacting with PRO is more reliable across all temperatures. In contrast, temperature-induced changes that may lead to different adsorption mechanisms are more likely to occur at higher surface coverages (i.e., higher *q*_*e*_ values). Thermodynamic parameters from partitioning model are in Table 5. Their signals agree with that from other models and suggest that entropy may play an important role in the adsorption process for the adsorption.

Chen et al.^65^ have shown that the estimation of thermodynamic parameters can vary substantially depending on the applied approach, as also observed here when comparing data from different models. For instance, the magnitude of Δ_ads_ *H* ^θ^ obtained from the partition model (23.5 kJ mol^–1^) is considerably lower than that derived from the Sips model (482 kJ mol^–1^). This pronounced discrepancy can be attributed to the variation of the heterogeneity factor *n* with temperature, which strongly influences the calculated thermodynamic quantities.

Overall, these results highlight that the thermodynamic parameters obtained are significantly model-dependent. Therefore, the proposed thermodynamic interpretation should be considered with caution, and the partitioning model is regarded here as a more suitable, although still approximate, representation under the conditions investigated. Considering the magnitude of Δ_ads_ *H* ^θ^ from partition model, the PRO adsorption process appears to be consistent with physisorption, in agreement with the mechanism proposed in Section 3.2.6.

#### 3.2.6 Adsorption Mechanisms

The removal of positively charged species by biosorbents is generally favored at pH values above the pH_PZC_, where the adsorbent surface and the adsorbate exhibit opposite charge signs, promoting electrostatic attraction. As discussed in Section 3.2.1, electrostatic interactions play a key role in the adsorption mechanism of PRO. For BP-ChCl/MSA, at pH 2, adsorption is hindered by electrostatic repulsion between the positively charged surface and the cationic PRO species. Nevertheless, the observed low removal (∼5%) indicates the contribution of additional interactions, suggesting a multimechanistic adsorption process.

At pH 4, the surface of BP-ChCl/MSA is close to its pH_PZC_ (4.5), resulting in low surface charge density. Under these conditions, nonelectrostatic interactions, such as hydrogen bonding, π–π interactions with aromatic domains, hydrophobic effects, and interactions with functional groups introduced by DES modification, are likely predominant. At pH 7, the surface becomes negatively charged (pH > pH_PZC_), favoring electrostatic attraction toward cationic PRO. While higher pH conditions favor electrostatic interactions, pH values close to the pH_PZC_ promote nonelectrostatic interactions, both contributing to the overall adsorption process.

Further evidence of the role of electrostatic interactions is provided by experiments conducted in the presence of salts (Section 3.2.7), where a significant decrease in PRO adsorption was observed. This behavior can be attributed to charge screening effects at higher ionic strength, which hinder electrostatic interactions, thereby confirming their important contribution, particularly at higher pH values.

To obtain more insights into the mechanisms of PRO adsorption, ATR–FTIR spectra of PRO and of the materials BP-MSA and BP-ChCl/MSA after PRO adsorption were obtained (Figure S6 in Supporting Information). In the PRO spectra, the band observed at 3272 cm^–1^ corresponds to the stretching vibration of the hydroxyl group (secondary alcohol), while the band at 2968 cm^–1^ is attributed to the stretching vibration of the C–H bond. The C=C stretching vibration of the aromatic ring appears at 1581 cm^–1^, and the bands at 1451 cm^–1^ and 1398 cm^–1^ are assigned to the in-plane bending vibration of O–H and the symmetric out-of-plane bending vibration of C–H, respectively.^72^ In the fingerprint region, the absorption at 1267 cm^–1^ is related to the asymmetric deformation of the C–O–C group from alkyl-aryl ethers, while the band at ∼1240 cm^–1^ can be attributed to C–N stretching vibrations, with possible contributions from C–O stretching modes. Another intense band at 1106 cm^–1^ is associated with the C–O–C stretching vibration of the aryl–alkyl ether linkage. Finally, the band at 771 cm^–1^ is attributed to the out-of-plane bending vibration of the naphthalene ring.^72^

In the BP-MSA + PRO and BP-ChCl/MSA + PRO spectra, the C–H and C–N stretching vibrations of the secondary amine are observed around ∼2950 cm^–1^ and ∼1260 cm^–1^, respectively. In addition, the C–O–C stretching vibration of the aryl-alkyl ether present in PRO is also detected. For the BP-ChCl/MSA + PRO system, the bending vibration of the naphthalene ring is clearly observed. These results support the presence of PRO on the adsorbent surface.

Notable shifts in characteristic bands were observed in the spectra of BP-MSA and BP-ChCl/MSA after PRO adsorption (Table S1 shows specific bands for each material, before and after adsorption), indicating distinct interaction mechanisms for these materials. In BP-MSA, a pronounced shift of the carbonyl band from 1731 to 1703 cm^–1^, accompanied by a slight redshift of the O–H/N–H stretching region (3373 cm^–1^ → 3367 cm^–1^), reveals the formation of strong hydrogen-bonding interactions between the carbonyl groups on the adsorbent and the hydroxyl and/or secondary amine functionalities of PRO.^73^ In contrast, BP-ChCl/MSA exhibits negligible changes in the carbonyl region but a pronounced change in the 1200–1000 cm^–1^ range, assigned to C–O/C–N vibrations modes, indicating that adsorption in this system can be governed primarily by interactions involving polar groups exposed by the DES modification, likely engaging the ether oxygen or amino group of PRO through hydrogen bonding or dipole–dipole interactions, with possible contributions from proton-mediated processes. Altogether, these results suggest that the two materials may provide distinct active sites, with carbonyl–driven interactions prevailing in BP-MSA, whereas DES-derived polar groups dominate the adsorption behavior of BP-ChCl/MSA.

In the absence of more surface-sensitive characterization techniques (e.g., XPS or Raman spectroscopy), the interpretations in this section should be regarded as plausible mechanistic hypotheses rather than definitive conclusions.

#### 3.2.7 Reuse and Adsorption Selectivity

The reusability tests of BP-ChCl/MSA for PRO adsorption are shown in Figure 9a. During the first and second cycles, the material retained a high adsorption efficiency (%R ≈ 95%), indicating good structural stability and strong affinity of the active sites for PRO. In the third cycle onward, a significant decrease was observed, with the %RE dropping to approximately 80% in the third cycle and to around 45% in the fourth.

**Figure 9:**
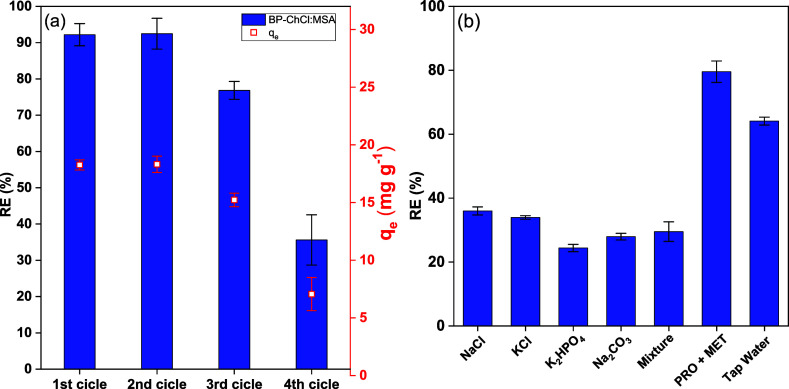
(a) Reusability of BP-ChCl/MSA after four cycles of PRO adsorption. Adsorption conditions: 10.0 mg of material +10.00 mL of PRO solution (20.0 mg·L^–1^) at pH 7, stirred at 100 rpm for 24 h at 25 °C. Desorption conditions: 10.00 mL of ethanol at 96%, stirred at 100 rpm for 24 h. (b) PRO removal using BP-ChCl/MSA in the presence of individual salts (NaCl, KCl, K_2_HPO_4_, Na_2_CO_3_ at 0.1% m v^–1^), their mixture, MET, and tap water. Adsorption conditions: 10.0 mg of adsorbent and 10.00 mL of PRO solution (20.0 mg L^–1^) at pH 7, under agitation at 100 rpm for 24 h at 25 °C.

The gradual reduction in %RE after the second cycle can be attributed to partial saturation or irreversible occupation of adsorption sites, possibly due to strong interactions between PRO molecules and the functional groups of the BP-ChCl/MSA surface. Additionally, pore blockage and loss of surface functional integrity during regeneration may contribute to the reduced adsorption capacity. The modification using ChCl/MSA DES likely enhances the initial adsorption by providing hydrogen-bonding and π–π interactions with PRO; however, these same interactions may also hinder complete desorption, resulting in cumulative deactivation of active sites.

Overall, BP-ChCl/MSA exhibited good recyclability for up to two cycles of desorption with minimal performance loss, but the subsequent cycle showed a noticeable decrease in the adsorption capacity. This behavior is consistent with other biochar-based or DES-modified adsorbents reported in the literature, where partial site deactivation and structural changes limit long-term reuse.

The selectivity of BP-ChCl/MSA toward PRO removal was evaluated in the presence of NaCl, KCl, K_2_HPO_4_, and Na_2_CO_3_ at 0.1% (m v^–1^), either individually or as a mixed salt system (Figure 9b). Compared with single-solute conditions (%RE ≈ 95%, first cycle in Figure 9a), the presence of inorganic ions led to a decrease in efficiency, with %RE values ranging from 25% to 37%, indicating significant interference with PRO adsorption. This behavior can be reasonably attributed to (i) competitive adsorption of ions for active sites and (ii) electrostatic screening effects, which may weaken the interaction between positively charged PRO molecules and negatively charged surface functional groups of BP-ChCl/MSA, as discussed in Sections 3.2.1 and 3.2.6. Notably, monovalent cations (Na^+^ and K^+^) produced a less pronounced decrease in %RE, suggesting partial screening of outer-sphere electrostatic interactions, in contrast to the stronger effects observed for the other ions. These results corroborate that the adsorption mechanism is likely not governed solely by electrostatic interactions but also involves nonelectrostatic contributions, such as hydrogen bonding, π–π interactions, and hydrophobic effects (Section 3.2.6).

It is important to highlight that the salt concentrations investigated here exceed those typically found in natural waters; therefore, higher %RE values are expected under environmentally relevant conditions. This was confirmed by experiments conducted in tap water, where a relatively high %RE (≈65%) was maintained, demonstrating the robustness of the adsorbent in more realistic matrices. These findings indicate that BP-ChCl/MSA is a promising candidate for wastewater treatment applications, particularly under conditions of moderate ionic strength.

In the presence of MET, a higher %RE (≈80%) was observed, indicating selective adsorption even in the presence of coexisting pharmaceutical compounds. The adsorption of PRO in the presence of TN was not evaluated due to overlapping absorption bands, which hindered accurate quantification by UV–vis spectroscopy. Future studies should focus on experiments with real wastewater matrices containing multiple pharmaceuticals to further assess and optimize PRO removal performance.

#### 3.2.8 Cost Analysis

A preliminary cost assessment at the laboratory scale was conducted to examine the economic feasibility of the modified biosorbents. The main cost contributions arise from the market prices of the key reagents, MSA (US$ 3.06 kg^–1^) and ChCl (US$ 6.11 kg^–1^), as well as operational expenses associated with water and electricity consumption for heating and drying steps (See Table S2, Supporting Information). Biosorbents are often regarded as low-cost materials due to the abundance and diversity of available biomass.^74^ The BP exhibited a low production cost of approximately US$ 2.16 kg^–1^, reflecting its simple preparation procedure, which mainly involves drying. In contrast, the BP-MSA showed a higher estimated cost of US$ 20.96 kg^–1^, largely due to the consumption of MSA (≈1.63 kg per kg of biosorbent) and the energy demand of the 24 h reflux process. The BP-ChCl/MSA presented the highest estimated cost (US$ 30.54 kg^–1^), primarily driven by the combined use of ChCl (≈1.78 kg per kg of biosorbent) and MSA (≈1.22 kg per kg of biosorbent), which dominate the overall expenses. Water consumption, estimated at 12 L per batch, had a negligible impact on the total cost, indicating that reagent and energy consumption are the principal economic drivers.

It should be emphasized that these values are approximate and based on laboratory-scale conditions. Several relevant factors were not considered in this estimation, including feedstock logistics and preprocessing, reactor design and scalability, reagent recovery and reuse, equipment and maintenance costs, transportation, labor, waste management, and detailed energy consumption. Despite this, the sustainable use of waste biomass, combined with the potential for DES recovery and process optimization, suggests that the costs could be reduced in commercial-scale production, reinforcing the potential of these materials for practical applications.

Reported production costs for biosorbents vary widely depending on the precursor and modification method, typically ranging from a few USD per kg for raw or minimally processed biomass (e.g., ∼0.05 USD kg^–1^)^75^ to significantly higher values when chemical treatments are involved.^76^ These variations highlight that the economic feasibility of biosorbents is strongly influenced by the extent of chemical modification and energy demand.

## 4 Conclusions

This study shows that treatment of BP with the ChCl/MSA DES enhances its ability to remove PRO from aqueous solutions. FTIR and TGA analyses indicate substantial chemical restructuring of the biomass after DES treatment, including extensive solubilization of polysaccharidic fractions, leading to a relative increase in lignin content. Partial hydrolysis was observed for the MSA-treated material (BP-MSA), but the DES produced significantly stronger synergistic modifications, suggesting distinct solvent–biomass interaction pathways. Whereas MSA acts primarily through acid-catalyzed hydrolysis and depolymerization of polysaccharides, the addition of ChCl provides a complementary hydrogen-bond acceptor (HBA) component that facilitates hemicellulose dissolution and more pronounced structural reorganization. These explain the superior performance of BP-ChCl/MSA in PRO removal. As evidenced by SEM, after DES treatment, the biopolymer matrix broke down into fine particles, exposing a higher surface density of functional groups, mainly from lignin, available to interact with PRO. The adsorption of PRO onto BP-MSA and BP-ChCl/MSA was best described by the PSO kinetic model. Notably, BP-ChCl/MSA exhibited a slightly higher rate constant than BP-MSA, indicating that the DES treatment enhanced the adsorption kinetics. Furthermore, the Sips isotherm model provided the best fit, showing maximum PRO adsorption capacities of 238.1 mg g^–1^ (BP-MSA) and 284.6 mg g^–1^ (BP-ChCl/MSA), respectively. These results demonstrate that the chemical and physical modification of BP through DES treatment enhances the availability of functional groups of lignin, which may promote interactions with PRO via electrostatic attraction, π–π stacking and hydrogen bonding. From a thermodynamic perspective, the estimated thermodynamic parameters showed significant dependence on the equilibrium model applied. The partitioning model provided a more consistent description of the system, suggesting that the adsorption process may be predominantly governed by physical interactions. Furthermore, the BP-ChCl/MSA biomaterial exhibited higher performance in reusability assays, maintaining high %RE in the first two cycles (up to 95%), and approximately 80% in the third cycle. These results suggested that the use of the DES ChCl/MSA to modify BP represents an effective, simple, and environmentally friendly strategy to enhance its adsorption capacity toward model pharmaceuticals such as PRO. While the present study focuses on ripe BP (*M. acuminata*, Cavendish type), the modification approach targets structural features common to lignocellulosic biomass, suggesting potential applicability to BPs from other cultivars and ripening stages. However, variations in composition associated with cultivar, origin, and maturation may influence the adsorption performance. Therefore, the present study should be regarded as a proof-of-concept demonstrating the effectiveness of the proposed modification strategy.

## Supplementary Material



## Data Availability

The data that support the findings of this study are available within the article and its Supporting Information.

## Notes

Hydrolysis mechanism of cellulose by the MSA effect; Intermolecular hydrogen-bond network between glucose units in cellulose, illustrating the strong chain–chain interactions that contribute to its high structural stability; Representation of the possible interactions between the deep eutectic solvent (choline chloride–based) and cellulose chains, illustrating the molecular mechanisms underlying the observed solubility; ΔpH versus initial pH (pH_0_) for pH_PZC_ determination of B-MSA and BP-ChCl/MSA; Sips and Langmuir fittings for adsorption of PRO on BP-ChCl/MSA at different temperatures; ATR-FTIR spectra of: (a) PRO, (b) B-MSA + PRO and (c) BP-ChCl/MSA + PRO; Laboratory-scale cost estimation for biosorbent production (PDF)
