# Supplementary material for: Banana Peel Modified with Deep Eutectic Solvents for Pharmaceutical Water Remediation
Source: ACS Omega. 2026 Jun 3;11(23):34226–44. doi: 10.1021/acsomega.6c01163 (PMC13280878; doi:10.1021/acsomega.6c01163)
Supplement: Supplementary file 1 [file ao6c01163_si_001.pdf]

BANANA PEEL MODIFIED WITH DEEP EUTECTIC SOLVENTS FOR PHARMACEUTICAL WATER REMEDIATION

Gabriela Chamorro Gil[1], João Antonio Tavares Barboza[2], Sérgio Scherrer Thomasi[2], Guilherme Max Dias Ferreira[2]*, Andrés Felipe Chamorro[3]*

[1]Research Group of Electrochemistry and Environment (GIEMA), Faculty of Basic Sciences, Universidad Santiago de Cali, Cali 760035, Colombia

[2]Group of Materials, Interface, and Solutions (MatIS), Department of Chemistry, Federal University of Lavras, Campus Universitário, Lavras, MG 37200-900, Brazil.

[3]Department of Chemistry, Faculty of Basic and Applied Sciences, Universidad Militar Nueva Granada, Cajicá 250247, Colombia.

Corresponding authors:
*Corresponding author: E-mail address - guilherme.ferreira@ufla.br;
Orcid: https://orcid.org/0000-0002-4762-2777; Phone: +55 35 38299797

* Corresponding author: E-mail address – andres.chamorro@unimilitar.edu.co;
Orcid: https://orcid.org/0000-0001-6209-4688; Phone: +57 (601) 6500000

Supplementary Materials

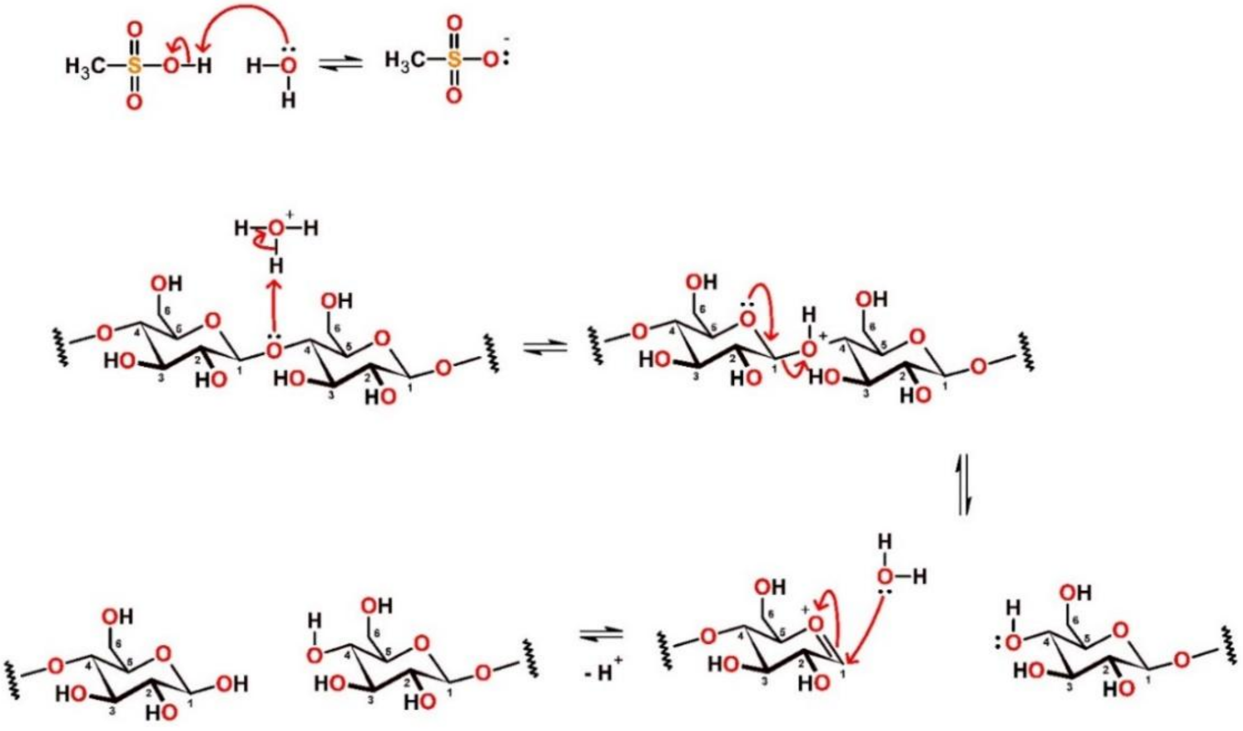

Figure S1. Hydrolysis mechanism of cellulose by the MSA effect.

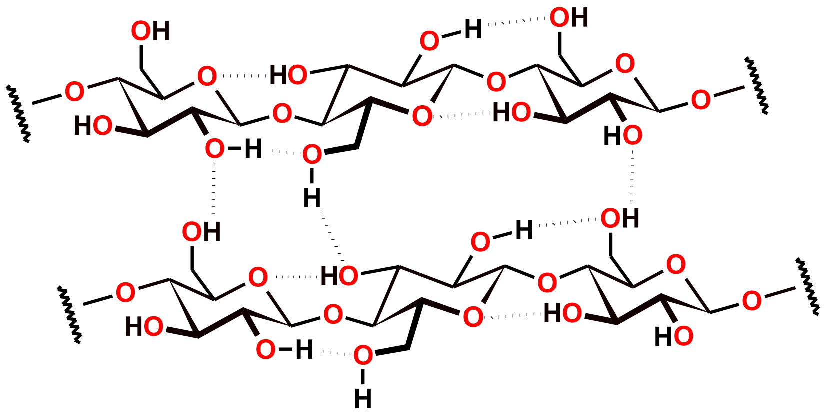

Figure S2. Intermolecular hydrogen-bond network between glucose units in cellulose, illustrating the strong chain–chain interactions that contribute to its high structural stability.

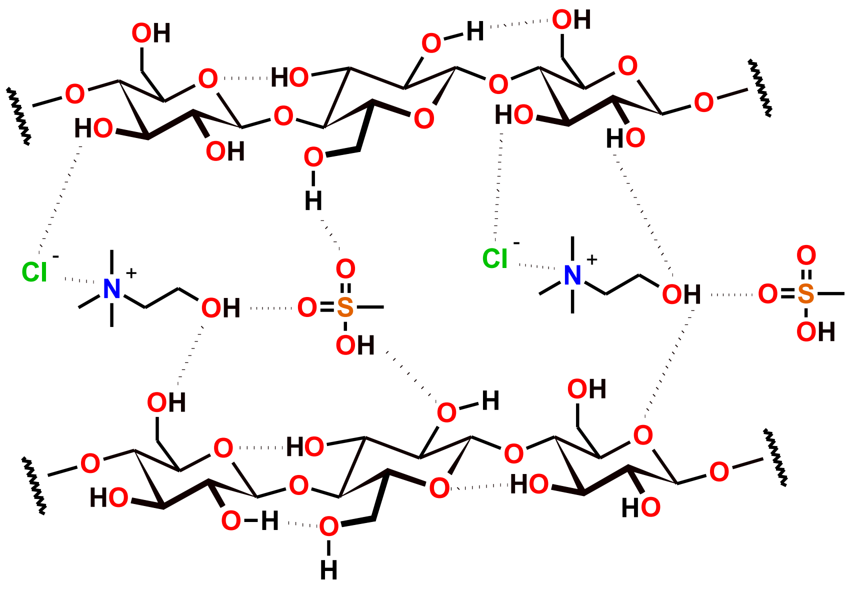

Figure S3. Representation of the possible interactions between the deep eutectic solvent (choline chloride–based) and cellulose chains, illustrating the molecular mechanisms underlying the observed solubility**.**

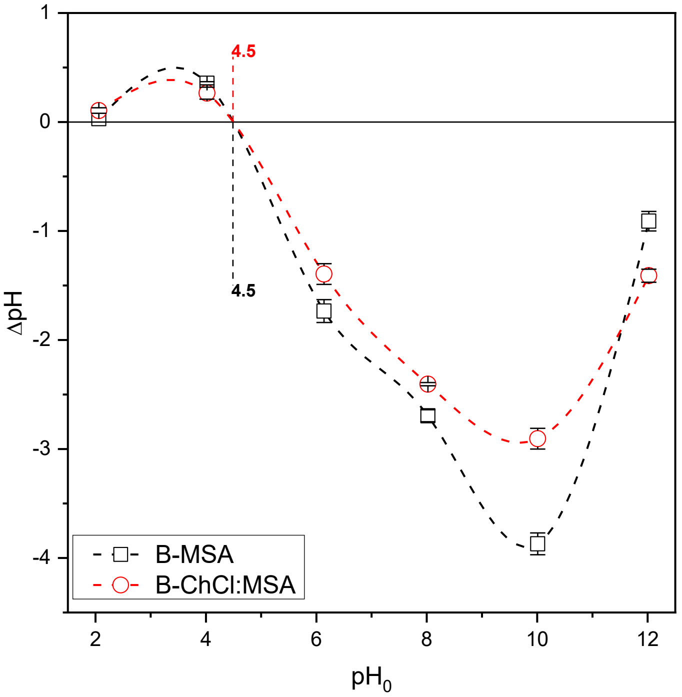

Figure S4. ΔpH versus initial pH (pH_0) for pH_{PZC} determination of B-MSA and BP-ChCl:MSA.

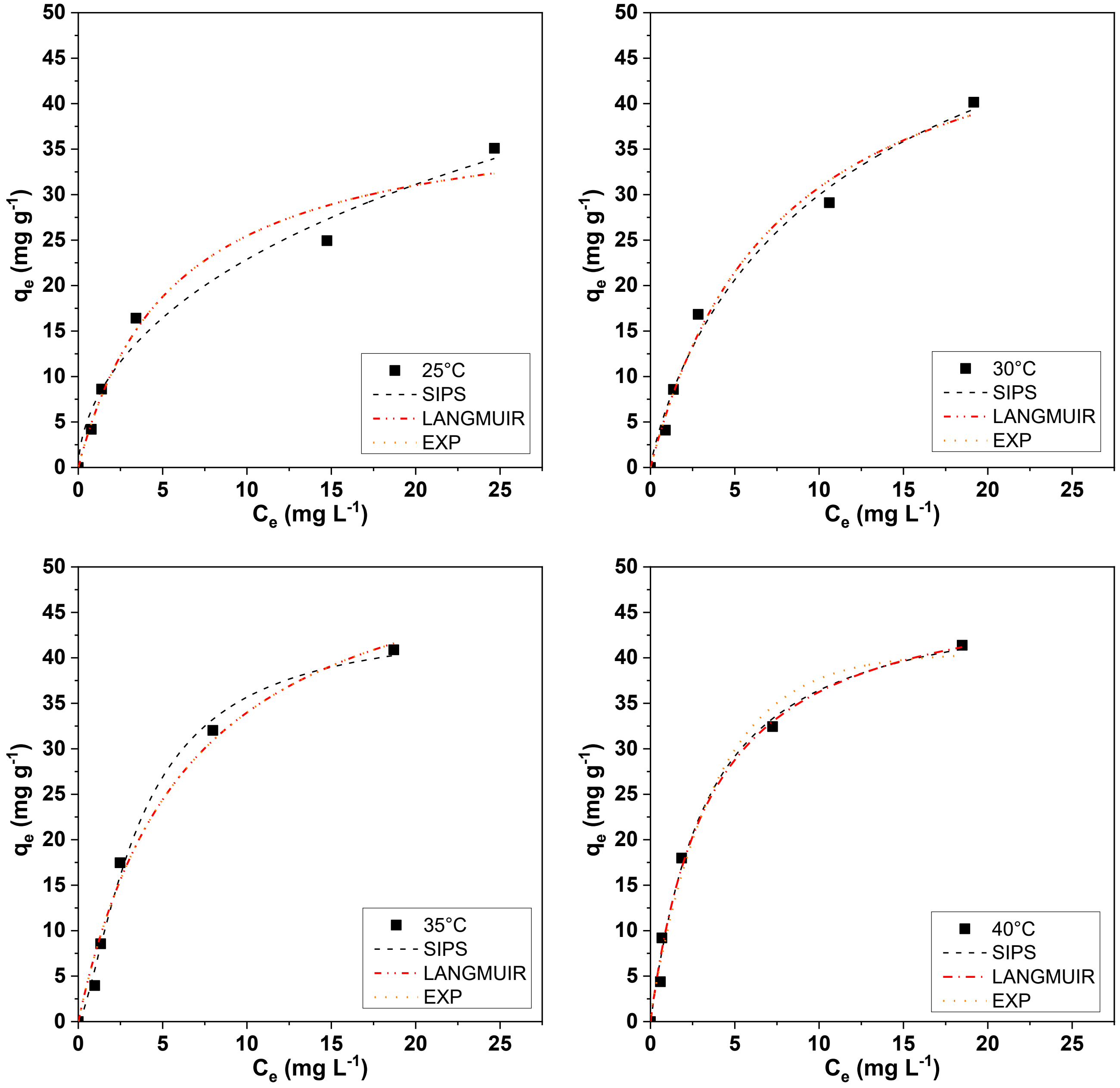

Figure S5. Sips, Langmuir and exponential fittings for adsorption of PRO on BP-ChCl:MSA at different temperatures.

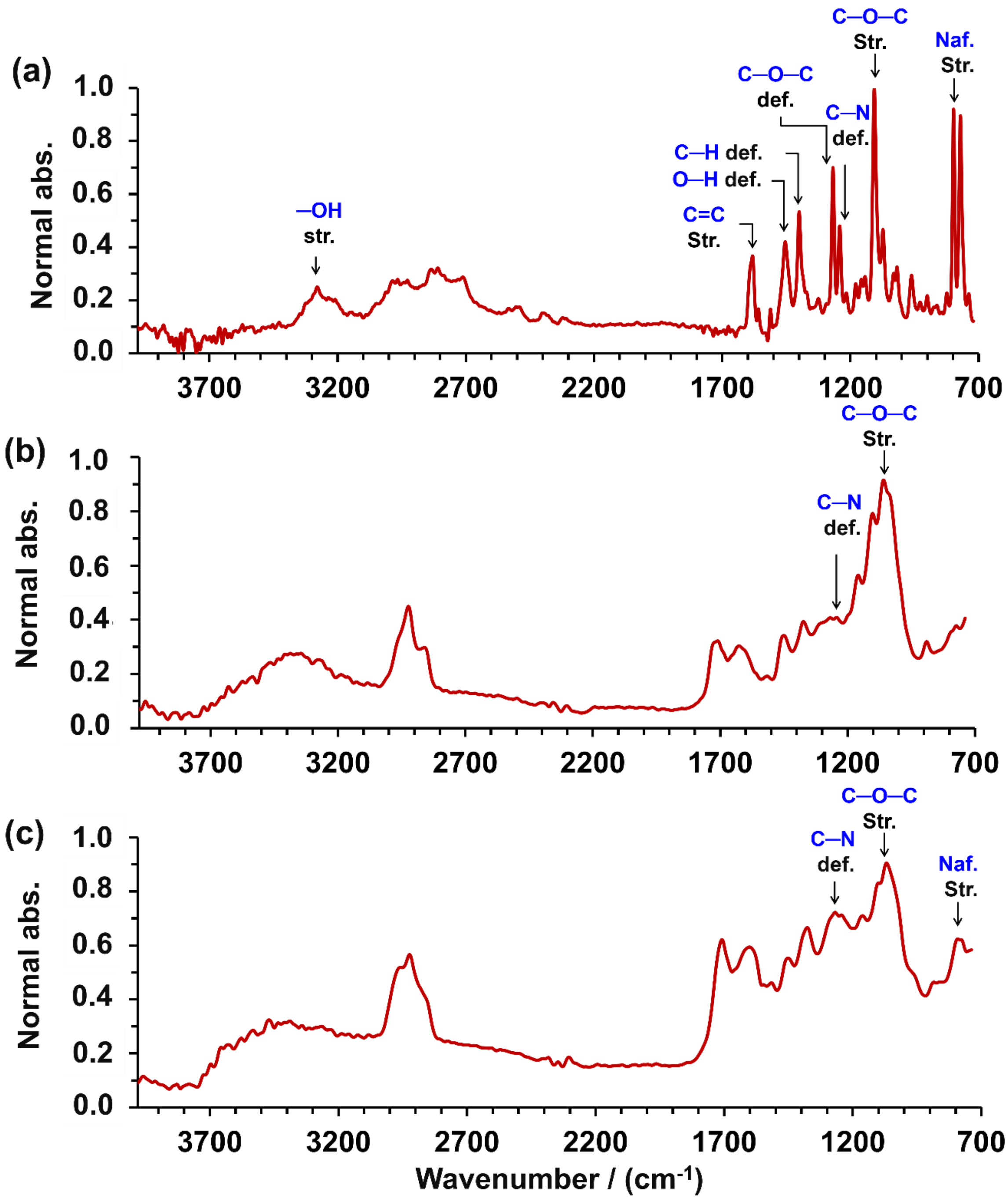

Figure S6. ATR-FTIR spectra of: (a) PRO, (b) B-MSA + PRO and (c) BP-ChCl:MSA + PRO.

Table S1. FTIR absorption bands of biomaterials.

	Material / Wavenumber (cm^{-1})				
Vibrations	BP	BP-MSA	BP-ChCl:MSA	BP-MSA + PRO	BP-ChCl:MSA + PRO
Str. -OH	3325	3373	3380	3367	3383
Str. -CH-	2917	2919	2916	2918	2916
Str. $-CH_2-$	2847	2849	2846	2852	2858
Str. C=O	1732	1731	1703	1703	1707
Def. C=C	1591	1610	1596	1612	1591
Str. C-O-C	1030	1055	1182	1058	1060

Str.= stretching; Def.= deformation

Table S2 - Cost estimation for producing 1 kg of B-MSA and BP-ChCl:MSA based on laboratory-scale experiments

Step	Subsections	Cost break up	BP-MSA (US$) Kg^{-1}	BP-ChCl:MSA (US$) Kg^{-1}
Biomass preparation	Drying process of the raw material	72 h x 1.1 kW x 0.15 kWh^{-1}	11.88	11.88
Biomass modification	Methanesulfonic acid	1.63 Kg x 3.06 US$ Kg^{-1}	4.99	-
DES modification	Methanesulfonic acid	1.22 Kg x 3.06 US$ Kg^{-1}	-	3.73
	Choline chloride	1.77 x 3.06 US$ Kg^{-1}	-	10.85
Biosorbent finalization	Washing cost	12 L x 0,01 US$ L^{-1}	0.12	0.12
	Drying of the biosorbent	24 h x 1.1 kW x 0.15 kWh^{-1}	3.96	3.96
		Total	20.95	30.54
